# A framework for multi-scale intervention modeling: virtual cohorts, virtual clinical trials, and model-to-model comparisons

**DOI:** 10.3389/fsysb.2023.1283341

**Published:** 2024-01-22

**Authors:** Christian T. Michael, Sayed Ahmad Almohri, Jennifer J. Linderman, Denise E. Kirschner

**Affiliations:** ^1^ Department of Microbiology and Immunology, University of Michigan–Michigan Medicine, Ann Arbor, MI, United States; ^2^ Department of Chemical Engineering, University of Michigan, Ann Arbor, MI, United States

**Keywords:** model study design, digital partners, disease modeling, tuberculosis, computational biology, pharmacokinetic-pharmacodynamic model, sensitivity analysis, agent-based model

## Abstract

Computational models of disease progression have been constructed for a myriad of pathologies. Typically, the conceptual implementation for pathology-related *in silico* intervention studies has been ad hoc and similar in design to experimental studies. We introduce a multi-scale interventional design (MID) framework toward two key goals: tracking of disease dynamics from within-body to patient to population scale; and tracking impact(s) of interventions across these same spatial scales. Our MID framework prioritizes investigation of impact on individual patients within virtual pre-clinical trials, instead of replicating the design of experimental studies. We apply a MID framework to develop, organize, and analyze a cohort of virtual patients for the study of tuberculosis (TB) as an example disease. For this study, we use *HostSim*: our next-generation whole patient-scale computational model of individuals infected with *Mycobacterium tuberculosis*. *HostSim* captures infection within lungs by tracking multiple granulomas, together with dynamics occurring with blood and lymph node compartments, the compartments involved during pulmonary TB. We extend *HostSim* to include a simple drug intervention as an example of our approach and use our MID framework to quantify the impact of treatment at cellular and tissue (granuloma), patient (lungs, lymph nodes and blood), and population scales. Sensitivity analyses allow us to determine which features of virtual patients are the strongest predictors of intervention efficacy across scales. These insights allow us to identify patient-heterogeneous mechanisms that drive outcomes across scales.

## 1 Introduction

Understanding the effectiveness of intervention measures in the context of patient-to-patient variability is a challenge in both drug and vaccine studies. Diseases such as cancer and infections such as COVID-19 and tuberculosis (TB) show patient variation in both infection outcomes and intervention efficacies. Actionable data–data that may help us determine efficacious interventions as well as understand patient variability–is limited by the frequency of patient visits, the quantity and quality of patient data, monitoring procedures, and resources.

Computational models are an additional approach toward gaining valuable insights into disease and accompanying interventions. Models applied in biomedicine have been used to disentangle the multitude of interconnected components of large complex systems such as cancer, HIV-1/AIDS, influenza and TB. Many modeling studies seek to: i) replicate experimental *in vivo, in vitro,* or *in situ* studies by using *in silico* experiments while maintaining experimental design, such as experimental interventional studies (Aggarwal and Ranganathan, 2019); ii) determine mechanistic impacts of model components and perturbations/treatments/interventions on output, e.g., by using sensitivity analyses; and/or iii) develop model extensions or reductions to determine the relative importance of detailed components (Kirschner et al., 2014).

In order for a model to credibly perform credible *in silico* experiments requires rigorous validation against available data (Tatka et al., 2023). The precision and rigor required are system-specific and adapted to the expected use of the model’s output (Fogarty et al., 2022), and consequences of incorrect model predictions (Aldieri et al., 2023). Various standards exist to codify model validation (Fogarty et al., 2022; Tatka et al., 2023); including the ten rules for model credibility developed by the Multi-scale Modeling Consortium (Erdemir et al., 2020; Fogarty et al., 2022; Nanda et al., 2023; Tatka et al., 2023) for systems biology approaches, as well as the ASME VandV40 standards (ASME, 2018; Aldieri et al., 2023; Tatka et al., 2023), and NASA standards for models and simulation (NASA, 2016; Tatka et al., 2023). Each of these standards establishes a series of assessments by which we can establish the appropriateness of a model to address a given question of interest relative to the model’s context of use. Here we describe a framework for using a validated computational model, for example, in a virtual clinical trial.

When we design virtual clinical trials from computational models, we find one luxury in that the definition of a “virtual patient” is flexible. For example, if a pharmacokinetic-pharmacodynamic (PK-PD) model is being implemented, then a patient’s pharmacokinetic identity is entirely defined by a set of PK-PD parameters. In many individual-scale computational approaches, every population generated by a model is independent, which reflects the design that motivates experimental interventional studies. However, that same virtual patient can serve in multiple “what-if” scenarios, such as determining effects of model stochasticity or perturbed biological influences or as a negative control (no drug treatment). The experimental analogue to this approach would be tantamount to running different experimental interventions on the same patient under the same conditions and scenarios.

With our ability to select amongst many types of models that can credibly represent the same system, we need a methodology to compare models in an implementation-agnostic way. We have seen a recent push to standardize modeling approaches with modeling ecosystems such as CompuCell3D (Poplawski et al., 2008; Shirinifard et al., 2009), VCell (Blinov et al., 2008; Schaff et al., 2016), PhysiCell (Ghaffarizadeh et al., 2018), as well as standardized language for ODE model implementation such as SBML (Keating et al., 2020), SED-ML (Bergmann et al., 2017; Smith et al., 2021), COMBINE, OMEX (Bergmann et al., 2014; Neal et al., 2020), and others (Tatka et al., 2023). With this variety of platforms, software, computational frameworks, and databases available (computational models, medical digital twins, etc.), it is likely impossible to develop a single computational package to automate analysis or comparison methodologies that account for the myriad of modeling approaches possible without overly constraining their use context. One component common to all models is the representation a real patient by a virtual one (with varying degrees of accuracy and refinement), hence we can create a broadly-applicable methodological framework to perform model-to-model comparisons.

**FIGURE 1:**
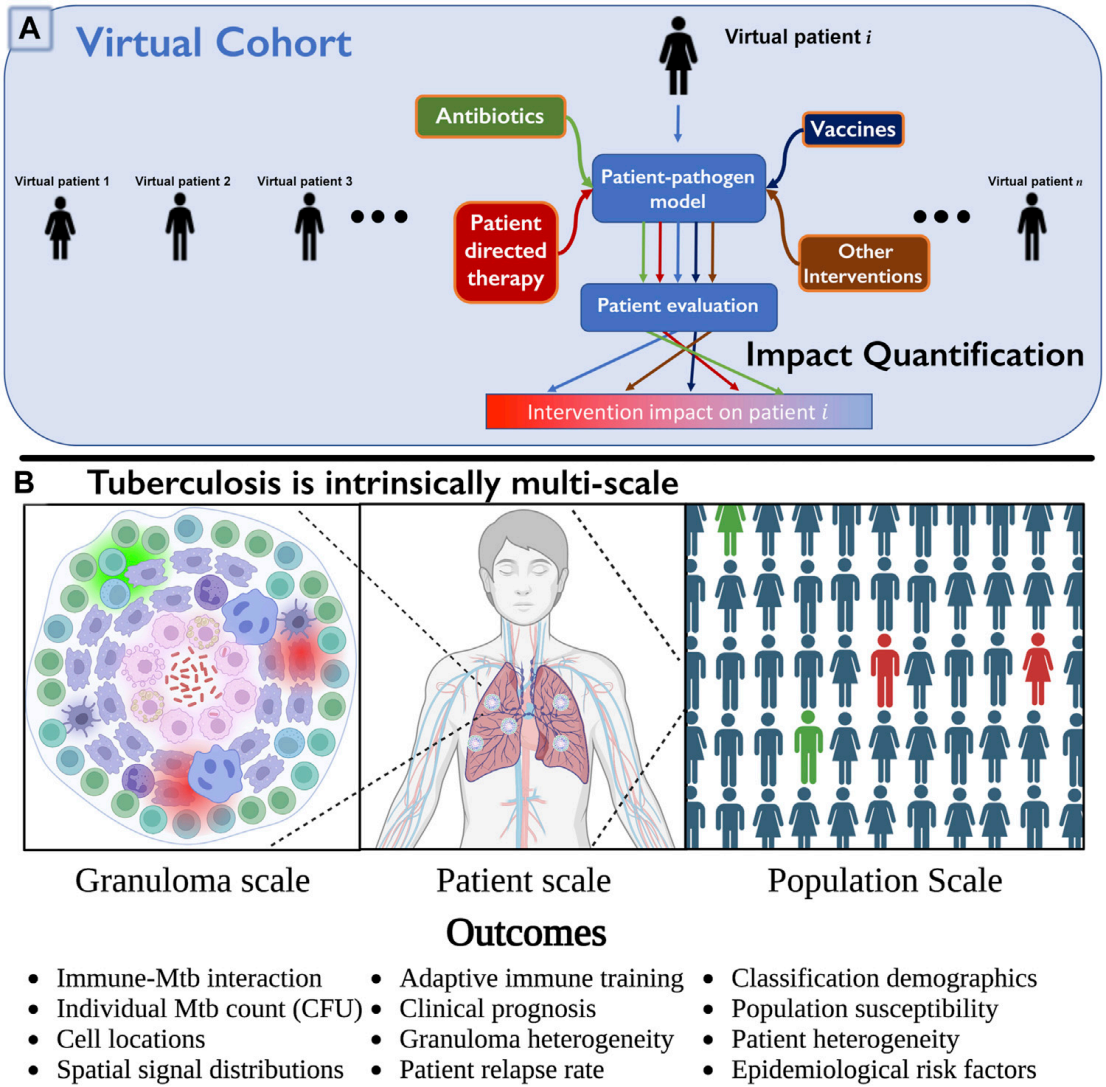
**(A)** Multi-scale intervention design to study over three physiological scales. We include a collection of virtual patients, a virtual cohort, that can each be represented by a control model or represented under various interventions applied (e.g., *HostSim* and a perturbed version, such as with drugs or vaccines). The virtual patient can be evaluated in each scenario, and impact level quantified by observing differences in specific patient outcomes. This can be quickly repeated for many patients in parallel to determine an overall population-scale impact (cohort effect), or to examine which subpopulations respond to interventions. **(B)** We illustrate three of the operative scales critical to understand TB. Lung granulomas encompass the complex dynamics of *Mycobacterium tuberculosis* (Mtb) populations and their interactions with various lymphocyte populations. Clinical classification of the patient (active or latent disease) is determined by multiple granulomas interacting with the patient’s lymphatic system. At the population scale, patients within a cohort vary in their susceptibility to infection and response to treatment, complicating our understanding and prediction of the demographic of clinical classifications. Note: we created Panel **(B)** using BioRender.com.

In this work we propose a generally applicable methodological framework, which we refer to as a *multi-scale interventional design* (MID) framework: a method of developing a cohort of virtual patients that we use to examine impacts of interventions on each virtual patient within a virtual cohort by tracking dynamics across physiological scales, from within-patient, through whole-patient, and up to the population scale (Figure 1A). Using a MID framework requires three key components: i) a cohort of *virtual patients*, along with a biological justification as to why the *same virtual patient* is able to be represented in multiple models; ii) a set of two related and validated *model versions*, such as a control model and an experimental model if representing, for example, a treatment intervention; and iii) an *impact quantification method* by which the outcomes of both model versions can be meaningfully compared.

Consider TB, a disease caused by an infectious bacterium *Mycobacterium tuberculosis* (Mtb) that has infected one-fourth of the current world’s population (WHO, 2022). In 2020, TB had a comparable annual death-toll to COVID-19 (WHO, 2020), and concurrent infection with COVID-19 or HIV has increased mortality for TB patients (WHO, 2022). Patients infected with Mtb may eliminate infection, control infection (resulting in latent TB disease) or fail to control infection (resulting in active TB disease), yet the factors determining those outcomes are not fully understood. Note, it is important to distinguish that Mtb are the bacteria that cause infection, whereas tuberculosis (TB) is the disease that results from infection. Data for analysis of Mtb infection progression typically comes from low-resolution measurements in patients (e.g., sputum analysis (Portevin et al., 2014; Guzzetta et al., 2015; Esmail et al., 2016)) or at necropsy when studying non-human primates (NHPs) or other animal models (Barry et al., 2009; Martin et al., 2017; Lin and Flynn, 2018; Wong et al., 2020; Grant et al., 2022). As a result, deriving mechanistic insights to time-evolution of Mtb infections and its interplay with patient heterogeneity across populations is a crucial step in improving our ability to study TB as well as other diseases.

Pulmonary TB, the most common form of the disease, is a highly complex disease with multiple interacting systems determining patient fate (note that we will also refer to patients as hosts as this is common terminology for an infectious disease). There is heterogeneity in lung granulomas, the focal structures of Mtb-host interaction, within individual TB hosts that is critical to prediction of host outcomes (Cadena et al., 2017; Lyadova, 2017; Cicchese et al., 2020). Host-scale dynamics are also heterogeneous and fall into at least three groups that exist on a spectrum: hosts that will clear the infection, control the infection, or fail to control infection and thus suffer active disease (Lin and Flynn, 2018). The dynamics of Mtb infected cohorts are also heterogeneous, e.g., some hosts improve with drug treatment rapidly while others do not. Thus to understand host infection progression and treatment, it is imperative to study TB at multiple scales and decipher how small-scale interactions influence large-scale findings (Figure 1B), making it an ideal candidate to test the MID framework.

To demonstrate our ability to study virtual cohorts using a MID framework, we implemented and tested our framework on multiple versions of *HostSim*, our next-generation, within-host to whole-host scale computational model of Mtb infection. These versions include a negative control version of *HostSim*, wherein infection of virtual TB hosts is left untreated, as well as three simple drug intervention versions for comparison. We implemented and tested these drug interventions in our virtual cohort and demonstrated that MID is an effective framework type to yield multi-scale virtual patient insights on complex biological problems that both include and explain patient heterogeneity at each scale.

## 2 Methods

Creating a MID framework requires three interconnected components: 1) a virtual cohort $\left\{VH\right\}$ , 2) a pair of related model versions: a control model $M_{0}$ and an intervention model $M_{P}$ to represent these hosts, and 3) an impact quantification: a method of evaluating and comparing the projected trajectories and final states of the virtual hosts between model versions. We present these components in the context of TB as an example. We also describe an updated version of *HostSim*, our previously published model of a whole-host, which captures the immune response to infection with Mtb within 3 physiological compartments: lungs, lymph nodes, and blood that represent pulmonary TB.

### 2.1 Creating the virtual cohort - a collection of 500 virtual hosts, $\left\{VH\right\}$

In our virtual cohort, each virtual host represents a typical host infected with Mtb with no comorbidities, and our virtual cohort will be generated to well-represent the demographic range of untreated patient outcomes observed in the biological context. We give our virtual hosts as an infection inoculum, 13 founding Mtb and one to five resting macrophages on day 0. Our virtual hosts represent Mtb infection progression in individuals up to 400 days post-infection, tracking granuloma cellular and bacterial composition once per day. In practice, each virtual host ( $VH_{i}$ ) in the cohort ( $\left\{VH\right\}$ ) is recorded as a granuloma and whole-host scale parameter set $P_{i}$ that is preserved between all versions of that virtual host (whether disease, treatment, etc.), which we refer to as the *virtual patient (host) identity.* We choose our virtual cohort of 500 virtual hosts ( $\left.\left\{VH\right\}\right)$ such that we capture the demographic of clinical outcomes observed in reality (Cadena et al., 2017). We select these parameter values by using the Latin Hypercube Sampling (LHS) method to generate values within a biologically viable range that we calibrate to multiple datasets (Section 2.3), ensuring that we accurately capture the heterogeneous spectrum of host outcomes. Note that the LHS method of parameter selection promotes stochastic and stratified coverage of the parameter space under the assumption of uniform distribution of each parameter within the experimental ranges (Helton and Davis, 2003; Cacuci and Ionescu-Bujor, 2004; Marino et al., 2008).

### 2.2 TB virtual host model*: HostSim* as $M_{0}$

Briefly, the *HostSim* model is based on known biology of pulmonary TB. When inhaled, Mtb is phagocytosed by macrophages. These inactive macrophages are unable to fully digest Mtb, which slowly replicates inside of them. Eventually, the macrophage bursts after reaching a carrying capacity of internal Mtb, and the cycle continues. In part due to the slow Mtb replication rate, inflammatory signals and antigen presentation occurs more slowly - and in NHPs, the lymph nodes (LNs) show no metabolic activity until 2–4 weeks post-infection (Coleman et al., 2014; Ganchua et al., 2018; Ganchua et al., 2020). Multiple granulomas form, typically one for each Mtb colony forming unit (CFU) (i.e., an individual Mtb bacterium) that lands within the lung (Martin et al., 2017). Mtb-specific T-cells arrive from LNs to activate macrophages and allow them to destroy intracellular Mtb and induce apoptosis of infected macrophages. These dynamics result in the development of a complex structure called a granuloma that comprises Mtb, live immune cells, and dead tissue (caseum).

**FIGURE 2:**
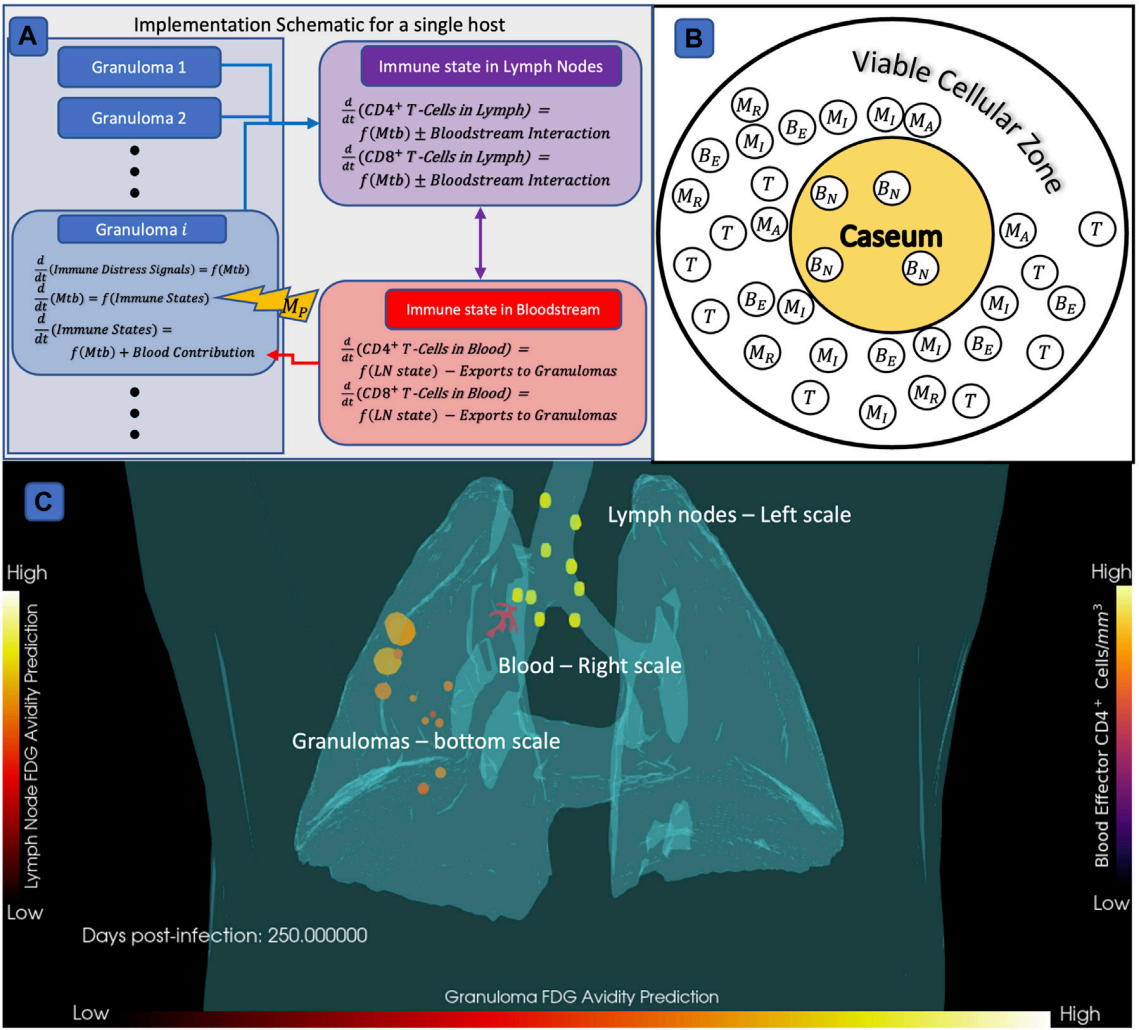
Diagram of *HostSim* model construction, $M_{0}$ . **(A)** Granulomas within lungs (blue compartments) are linked to lymph node (purple) and blood (red) compartments (details in Supplementary Material S2). We represent interventions as being applied to the equations governing Mtb development. **(B)** Diagram of a simplified granuloma as represented in *HostSim*. In the central caseum sub-compartment, nonreplicating bacteria are trapped within a hypoxic/necrotic core. All other species, including macrophages, T-cells, and extracellular bacteria, are in the viable cellular zone. Note that in *HostSim*, the viable cellular zone is treated as well-mixed for the sake of cell-cell interactions. **(C)** Lung granulomas and lymph nodes of virtual TB host at t = 250 days post-infection shown within the context of a lung and body triangulation of a nonhuman primate [courtesy of Henry J. Borish in JoAnne Flynn Lab, University of Pittsburgh]. Cylinders on the trachea represent the lymph node compartments, and spheres (colored by their CFU count and sized based on their cellular composition) represent granulomas. The branching blood vascular surface is colored based on blood effector CD4^+^ T-cell concentration. (Details of visualization are in Supplementary Material S1 Section 3).

*HostSim,* our untreated virtual host model, is a multi-scale computational model of an individual host that represents both the tissue-scale and whole-host scale response to pulmonary Mtb infection (Joslyn et al., 2022a; Joslyn et al., 2022b). We created a next-generation version of *HostSim* herein to include additional biological features and better capture Mtb infection immunobiology (see Supplementary Material S1 Section 2 for model updates, and Supplementary Material S2 for a complete model description and list of equations). We represent three physiological compartments in our hybrid computational model *HostSim*: lungs, LNs, and blood. The lung compartment captures a collection of lung granulomas represented as agents in an agent-based model. Each agent is itself comprised of a system of 22 nonlinear ordinary differential equations (ODEs) describing interactions between macrophages, three subpopulations of Mtb - intracellular, extracellular, and non-replicating; cytokine signals (e.g., IL-4, IL-10, IL-12, and TNF- α ), and different T-cells in various states of differentiation (Figure 2). Granulomas allow antigen-presenting cells to travel to LNs proportional to the Mtb burden within a granuloma, and the LN clonally expands Mtb-specific T-cells. T-cells are released from the LN compartment (described by ODEs) into blood (also represented by ODEs) where they may be recruited into lung granulomas. Since each granuloma has its own instantiation and parameterization within our ODE system, and formation of new granulomas makes the number of granuloma ODE trajectories variable, we consider *HostSim* to be a hybrid agent-based model. *HostSim* is simulated in MATLAB using the ode15s variable order ODE solver for time-stepping the ODE portions of *HostSim*.

When running simulations, cytokine signals and antigen presenting cells circulate to a virtual host’s LN compartment, which selectively clones Mtb-specific CD4^+^ and CD8^+^ T-cells. We have newly-calibrated parameter ranges to a variety of data from both NHPs (Gideon et al., 2015; Marino et al., 2016; Cadena et al., 2018; Darrah et al., 2019) and our fine-grained model of a single granuloma, *GranSim*, to capture the heterogeneity both between hosts and between granulomas within a single host (see Section 2.3).

### 2.3 Calibrating the virtual cohort $\left\{VH\right\}$ to be represented in $M_{0}$

We first need to calibrate *HostSim* in order for it to be a credible $M_{0}$ in our MID framework. With 201 varied parameters and 3 compartments, *HostSim* requires careful calibration that leverages known constraints and biological ranges. As in (Joslyn et al., 2022a; Joslyn et al., 2022b), we calibrate our model by comparing its outputs to data taken from 646 NHP granulomas assembled over the last 15 years (Gideon et al., 2015; Marino et al., 2016; Cadena et al., 2018; Darrah et al., 2019). We use our previously published calibration method, CaliPro (Joslyn et al., 2023), to refine both granuloma and LN parameter ranges from our previously calibrated values (Joslyn et al., 2022b). Our calibration criteria are implemented at the granuloma scale, and each criterion tests the proximity of simulated granulomas to granuloma data collected from NHPs (Gideon et al., 2015; Marino et al., 2016; Cadena et al., 2018; Darrah et al., 2019), as well as synthetic data from our fine-grain model of a single granuloma, *GranSim* (Segovia-Juarez et al., 2004; Pienaar et al., 2017; Warsinske et al., 2017; Sarathy et al., 2019; Cicchese et al., 2020; Budak et al., 2023).

Briefly, CaliPro is a calibration method that incorporates a broad range of model parameters and multiple and varied datasets. Using LHS, we choose a stratified collection of parameter values out of a broad parameter (Marino et al., 2008). CaliPro evaluates the model at each of these parameter values and determines whether the outputs are sufficiently close to the given dataset(s) to be admitted to a “pass set”, i.e., meeting heuristic criteria that suggest that model output is biologically relevant. CaliPro then shrinks the parameter ranges to exclusively capture the pass set while still covering the broadest possible set of parameters. This process is iterated multiple times. After calibration, 90% of granulomas “passed” all tests against calibration criteria, which are described in Supplementary Material S1 Section 2.2. Our calibrated parameter ranges are listed in Supplementary Material S2 Section 5.

### 2.4 Validation that our virtual cohort hosts capture population demographics for TB

Our goal is to use *HostSim* simulations to determine, on 3 physiological scales: population-scale, host-scale, and granuloma-scale, which features drive both granuloma and whole host infection outcomes. As such, our virtual cohort should reflect epidemiological contexts for TB (Joslyn et al., 2022b; WHO, 2022), and our use case of *HostSim* is to generate a collection of virtual hosts whose trajectories agree with distributions of available global data on humans for TB. To do this, we define virtual host classifications in a clinically interpretable way. For studying TB, our classifications are clinically latent, bacteria sterilizing, and active disease. We classify virtual hosts as having active TB if either 1) they have higher total lung CFU than an active-host cutoff of $3.2\cdot 10^{5}$ ; or 2) they have at least one granuloma which increases by more than 10% CFU between days 100 and 150 post-infection. We chose these times post-infection because primary infection sites have a transient peak of increasing bacterial numbers around day 30 before the immune system responds and forms a proper granuloma (Cadena et al., 2017). We define active disease based on data taken from 4 NHPs that were necropsied early due to severe Mtb infection (see Supplementary Material S1 Section 1, courtesy of the JoAnne Flynn lab). We classify virtual hosts as sterilizing hosts if their total lung CFU count has dropped to zero at or before 400 days post-infection. We consider all other hosts as having clinically latent TB, and there is a spectrum of outcomes within this group as is observed in humans and NHPs (Lin and Flynn, 2018). After performing calibration on the host and granuloma scales, our virtual cohort had a distribution of outcomes: approximately 90% of virtual hosts classified with latent TB, 6% with active TB, and 4% of virtual hosts sterilizing their infection entirely. This indicates that our virtual cohort reflects observed trends in patient outcomes at the population scale (Cadena et al., 2017; Lin and Flynn, 2018). Note our classifications are flexible as new data become available.

### 2.5 Developing intervention models $M_{P}$ for our virtual cohort

Our goal is to create a cohort that we can test different model perturbations such as antibiotic treatment, vaccines, or other interventions. To do this, our goal is to build versions of our model that represent a control version $M_{0}$ and an intervention version, $M_{P}$ . The intervention version should i) observe both the host and granuloma scale mechanisms from $M_{0}$ , and thereby maintains credibility, and ii) sufficiently represent intervention dynamics to identify key drivers of host-response. Here, in the interest of demonstrating the MID framework and its use, we use a highly-simplified model of antibiotic treatment of TB as an example of an intervention model $M_{P}$ . Our objective is to capture heterogeneity in the host-response to treatment over multiple physiological scales. To establish this approach, we use coarse-grained representation of 3 TB antibiotics, where we qualitatively represent the impact on bacterial burden in time by capturing the known modes of action of different antibiotics. These simplified TB drugs represent 3 different classes of drugs that are currently used to treat TB: isoniazid (INH), bedaquiline (BDQ), and pyrazinamide (PZA). While these drugs are typically used in combination therapy, here, for example, purposes, we implement each one individually. We model these drugs based only on their known killing (bactericidal) or bacteriostatic behaviors (Zhang and Mitchison, 2003; Jayaram et al., 2004; Sarathy et al., 2018; Budak et al., 2023), omitting for this simple model any consideration of pharmacokinetics or transport limitations in accessing portions of the granuloma as we have done in other work (Budak et al., 2023). We define an INH-like intervention version $M_{INH}$ , a PZA-like intervention $M_{PZA}$ , and a BDQ-like intervention $M_{BDQ}$ (each version representing an $M_{P}$ ). Here, we note some differences in these drugs’ mechanisms that we will phenomenologically capture: i) INH is able to penetrate into caseum and kill bacteria but is not taken up by infected macrophages (Jayaram et al., 2004; Prideaux et al., 2015; Nahid et al., 2016; Sarathy et al., 2016; Sarathy et al., 2018); ii) BDQ kills bacteria that it can reach more effectively but takes much longer to penetrate into caseum (Dhillon et al., 2010; Chahine et al., 2014; Prideaux et al., 2015; Sarathy et al., 2016; Sarathy et al., 2018); and iii) PZA is a bacteriostatic drug that slows bacterial replication but takes a long time to penetrate into caseum (Zhang and Mitchison, 2003; Prideaux et al., 2015; Nahid et al., 2016; Sarathy et al., 2016; Sarathy et al., 2018).

We represent dosing our virtual hosts by modifying the equations governing bacterial growth with the following unitless treatment values $A_{i}$ after intervention time t=200 days.

$$\frac{d}{dt}B_{E}=A_{1}\left(\text{Replication}\right)\pm \left(\text{conversion to }B_{I}\right)-A_{2}\left(\text{Death}\right)$$

$$\frac{d}{dt}B_{I}=A_{3}\left(\text{Replication}\right)\pm \left(\text{conversion to }B_{E},B_{N}\right)-A_{4}\left(\text{Death}\right)$$

$$\frac{d}{dt}B_{N}=\left(\text{conversion from }B_{I}\right)-A_{5}\left(\text{Death}\right)$$

where our control model $M_{0}$ *HostSim* is recovered if each $A_{i}=1$ . For $M_{BDQ}$ , we set $A_{2}=5\cdot 10^{7}$ , $A_{4}=5000$ and $A_{5}=10$ ; intervention parameters $A_{1}=1$ and $A_{3}=1$ since we do not treat BDQ as bacteriostatic. In $M_{INH}$ , we define these action coefficients relative to $M_{BDQ}$ - in $M_{INH}$ , we set $A_{2}=2500$ since INH is less effective at killing extracellular bacteria; we set $A_{5}=5$ since more INH ends up in caseum though it is less effective at a given concentration than BDQ, and $A_{4}=1$ because our simplified INH does not get taken into macrophages; for INH we also set $A_{1}=1$ and $A_{3}=1$ as it is not bacteriostatic. For $M_{PZA}$ , the bacteriostatic effect is captured by setting $A_{1}=A_{3}=0.5$ , halving bacterial replication rates for all hosts. We set $A_{2}=1$ , $A_{4}=1$ , and $A_{5}=1$ since PZA is not bactericidal. It is important to remember that the *virtual patient (host) identity* parameter values ( $P_{i}$ ) used to define the virtual cohort $\left\{VH\right\}$ are independent of $M_{0}$ and $M_{P}$ . By running simulations using either $M_{0}$ or $M_{P}$ with the same parameters $P_{i}$ and initial conditions - and thus each virtual host $VH_{i}$ - the entire virtual cohort can be represented in every model version, while the treatment values $A_{i}$ are preserved across the cohort.

### 2.6 Impact quantification method for our MID framework

The final component of our MID framework is an *impact quantification* method that directly quantifies and compares the impact of the intervention model versions $M_{INH}$ , $M_{PZA}$ , and $M_{BDQ}$ against the negative-treatment $M_{0}$ at multiple physiological scales. In principle, comparisons between virtual hosts and model versions may use any outcomes and measurements that may be relevant to the system under study. Importantly, the selection of impact quantification is implicitly related to the model’s question of interest and context of use, since models may have different levels of credibility depending on which outcome is being observed. The multi-scale component of a MID framework comes from comparing the outcome of $VH_{i}$ represented in $M_{P}$ (i.e., $M_{P}\left(VH_{i}\right)$ ) to $VH_{i}$ represented in $M_{0}$ (i.e., $M_{0}\left(VH_{i}\right)$ ) for each virtual patient in the virtual cohort. Here, we perform this quantification by directly comparing CFU counts between model versions over time. Since $M_{0}$ and $M_{P}$ have identically formatted and nonnegative outputs - time-series data of all *HostSim* variables computed once per day - the ratio of the outputs may be considered at all scales. On the host scale, we examine the ratio of total lung CFUs as

$$\text{Host Impact score}=H_{S}\left(t;\;VH_{i}\right)=\log\left(\frac{M_{0}\;CFU\left(VH_{i}\right)+1}{M_{P}\;CFU\left(VH_{i}\right)+1}\right)\left(t\right)$$ (1)

In this way, hosts with $H_{S}\approx 0$ have very little treatment effectiveness, $H_{S}>0$ have a positive influence on the system outcome, and $H_{S}<0$ have a deleterious effect. Capturing impact score over time informs many aspects of the score, including projected time until expected results of intervention. We can also compute an impact score at other physiological scales. For example, at the granuloma scale, we compute an impact score for each granuloma in the lung to obtain the granuloma impact,

$$\text{Granuloma Impact Score}=G_{S}\left(t;VH_{i}\right)=\log\left(\frac{M_{0}CFU\left(VH_{i}\right)+1}{M_{P}CFU\left(VH_{i}\right)+1}\right)\left(t\right).$$ (2)

### 2.7 Sensitivity analyses

As an additional form of impact quantification in a MID framework, we can also evaluate the impact of $M_{P}$ via *sensitivity analysis,* which allows us to identify parameters and initial conditions that drive specific features of model output. We use the partial rank correlation coefficient (PRCC) method, which is a computationally efficient and accurate method for performing sensitivity analysis on high-dimensional models (Marino et al., 2008; Renardy et al., 2019; Renardy et al., 2021). When given a set of model runs and a numerical output of that model, the PRCC method determines for each input parameter: i) a coefficient that measures the correlation between that parameter and the model output and ii) a p -value determining the statistical significance of that measurement. We typically use this method to understand the impact of parameter impacts on $M_{0}$ outputs. However, since the same virtual cohort $\left\{VH\right\}$ is being represented in both models, ( $M_{0}\left(\left\{VH\right\}\right)$ and $M_{P}\left(\left\{VH\right\}\right)$ ), sensitivity analysis methods apply to composite models $f\left[M_{0},M_{P}\right]\left(\left\{VH\right\}\right)$ . Since impact quantification methods such as expressions [1] and [2] satisfy the requirements of a composite model, we can perform sensitivity analysis on these scores as well to determine what patient characteristics correlate with intervention scores.

## 3 Results

### 3.1 Constructing a MID framework

If we run thousands of simulations, allowing for patient-to-patient variability and representation of each virtual host with and without interventions, we refer to our collection as a *virtual cohort*. We introduce our MID framework, our goal for which is to create an easily implementable layer for most computational modeling systems that represent individual patient dynamics. MID is a framework for making meaningful comparisons between the outcomes of individual virtual patients’ outcomes in between a negative control model $M_{0}$ and a perturbed *intervention version* of the model $M_{P}$ (see Figure 1A for a schematic).

To be specific, we require three interconnected components to create our MID framework, and they are: 1) a virtual cohort $\left\{VH\right\}$ , 2) a pair of related and validated models to represent those patients: a control model $M_{0}$ and an intervention model $M_{P}$ , and 3) a method of evaluating and comparing the projected trajectories and final states of the virtual hosts in either model version (see Section 2.6). One of the only model prerequisites is that there be a natural representation, or a biological justification, for how the same virtual patient $VH_{i}$ is represented by $M_{0}$ and $M_{P}$ . For example, if $M_{0}$ contains a simplified representation of pathogen replication time and $M_{P}$ contains a detailed pathogen life cycle interacting with a drug intervention, we must ensure that $M_{P}$ matches the “control limit” as the drug level approaches 0 . We must also use caution if two model versions have notably different representations of the same biological process. There must be some biologically-rooted justification as to why we can reasonably assume that the same host is being represented in both model versions.

Lastly, an impact quantification method should be specified that compares trajectories of individual virtual hosts represented in both the $M_{0}$ and $M_{P}$ versions in a biologically-interpretable manner. These should be specific to the particular model system and made to ensure that comparisons between the models are relevant to the intended goal of the intervention. For example, a drug intervention may have an outcome evaluation that weighs time to sterilization, pathogen burden, and drug toxicity. The MID framework components are simple enough that they can be applied to many models from multiple biomedical applications. We list a few examples of potential MID framework implementations in Table 1. Note that if we want to perform a MID framework study using highly stochastic models, we must take care in defining virtual host outcomes. For example, we might work with $\text{Mean}\left(M\right)$ and $\text{Var}\left(M\right)$ . Measurable features for impact quantification should be able to capture differences in dynamics between $M_{0}$ and $M_{P}$ at the scale at which the intervention is applied. As *HostSim* is deterministic (except for rare dissemination events) once the initial agent properties are defined, we omit such considerations from our TB application.

**TABLE 1:** Examples of potential application of the MID framework to biomedical systems. The virtual patient definition can be flexibly adapted and generalized to a broad set of virtual subjects and intervention types. Note that in all cases, $M_{0}$ and $M_{P}$ should be validated such that they may make credible claims about outcomes used in impact quantification. In some cases, finding a small impact may provide useful results (e.g., that a proposed treatment will not impact patient outcomes, or that a model simplification is sufficient to capture outcomes).

Model	Virtual cohort members	Model versions	Example impact quantification
*HostSim* (Joslyn et al., 2022a; Joslyn et al., 2022b)	Virtual host: a vector of parameters describing host PK/PD in each granuloma; Initial conditions of each granuloma and lymph node	*HostSim*, which encompasses all equations, dynamics, component interactions	Ratio of bacteria load between untreated host and host with antibiotic intervention for each granuloma and host; demographic of clinically Latent, Active, or Sterilizing patients
(Tuberculosis)		$M_{0}$ - No treatment	
		$M_{P}$ - With antibiotic treatment	
*Drug Interventions in GranSim* (Pienaar et al., 2017; Sarathy et al., 2019; Cicchese et al., 2020; Budak et al., 2023)	Virtual granuloma: vector of parameters for individual granuloma’s immune response; initial conditions and grid configuration	*GranSim*, which encompasses all agent probabilities, dynamics and cell behaviors, agent interactions	Function designating granulomas as controlling, non-controlling, or sterilizing as a function of their end state; expected bacterial counts by subpopulation
(Tuberculosis)		$M_{0}$ - No treatment	
		$M_{P}$ - With antibiotic treatment	
*Tuneable Resolution with GranSim* (Segovia-Juarez et al., 2004; Fallahi-Sichani et al., 2012a; Fallahi-Sichani et al., 2012b; Kirschner et al., 2014; Pienaar et al., 2016)	Virtual granuloma: vector of parameters for individual granuloma’s immune response; initial conditions and grid configuration	*GranSim*, which encompasses all agent probabilities, dynamics and cell behaviors, agent interactions	Function designating granulomas as controlling, non-controlling, or sterilizing as a function of their end state; the predicted growth phenotypes of bacteria and activation levels of immune cells
(Tuberculosis)		$M_{0}$ - Coarse grained representation of TNF- α , NF- κ B, or metabolism	
		$M_{P}$ - Fine grained representation of TNF- α , NF- κ B, or metabolism	
*Antibody-drug conjugate simulation* (Menezes et al., 2020; Menezes et al., 2022)	Virtual tumor: vector of parameters for individual tumor’s vascularization state, immune response, and initial grid conditions	Model that encompasses all agent probabilities, dynamics and cell behaviors, agent interactions	Function designating tumors as growing or shrinking as a function of structure and cancerous cell count
(Solid tumor)		$M_{0}$ - Untreated control	
		$M_{P}$ - Added antibody-drug conjugate treatment	
*Quantitative systems pharmacology simulation* (Norton and Popel, 2014)	Virtual patient: vector of parameters for virtual patient’s pharmacological parameters in the untreated case	Quantitative systems pharmacology simulation which describes immune-cancer interactions	Function quantifying the relative shrinkage of carcinoma with immune checkpoint inhibitors
(Hepatocellular carcinoma)		$M_{0}$ - Untreated control	
		$M_{P}$ - Added immune checkpoint inhibitors	
*Immune Response Agent-based Model* (Cockrell and An, 2017; Larie et al., 2021)	Virtual patient, wound, and environment: parameters determining of distributions of i) sustained endothelial tissue damage, ii) patient response thereto, iii) initial microstate, iv) external variables known to affect patient prognosis	Stochastic and mechanistic model of inflammatory response	Functions comparing the expectations, variances, and other descriptive distribution parameters of predicted oxygen deficit or cytokine levels in time with vs without treatment
(Sepsis)		$M_{0}$ - Untreated control model	
		$M_{P}$ - Model of proposed treatment or medical intervention for clinical sepsis	
*Fibrin contraction simulation* (Britton et al., 2019; Michael et al., 2023)	Virtual clot: collection of spatially arranged platelets embedded within a fibrin mesh	Subcellular element model that represents multiple platelets pulling on fibrin fibers to cause the emergent contraction of a blood clot	Function quantifying the relative amount of contraction of the blood clot and distribution of multi-platelet clusters
(*in vitro* Blood clot contraction)		$M_{0}$ - Untreated control	
		$M_{P}$ - Blebbistatin treatment to weaken contractile forces	

### 3.2 *HostSim* provides $M_{0}$ for MID to study TB over multiple scales

A key step of developing our MID framework study is to declare a control model, $M_{0}$ . This model represents the unperturbed system that we are interested in studying, which in our case is Mtb infection. We want this model to be well calibrated and validated, and to mechanistically represent our system at the scale that our intervention is going to perturb. For $M_{0}$ , we use an updated version of *HostSim*, our whole-host model of Mtb infection.

We update our TB simulation *HostSim* (Joslyn et al., 2022b) and recalibrate it to additional published datasets from NHPs across granuloma, host, and population scales (Gideon et al., 2015; Marino et al., 2016; Cadena et al., 2018; Darrah et al., 2019). We calibrated using our *CaliPro* procedure (Joslyn et al., 2023), integrating these data by using a population of 500 virtual hosts $\left\{VH\right\}$ sampled from within experimentally viable parameter ranges (see Section 2). Supplementary Figure S2 shows several state variable trajectories over time for a single representative virtual host with latent Mtb infection.

**FIGURE 3:**
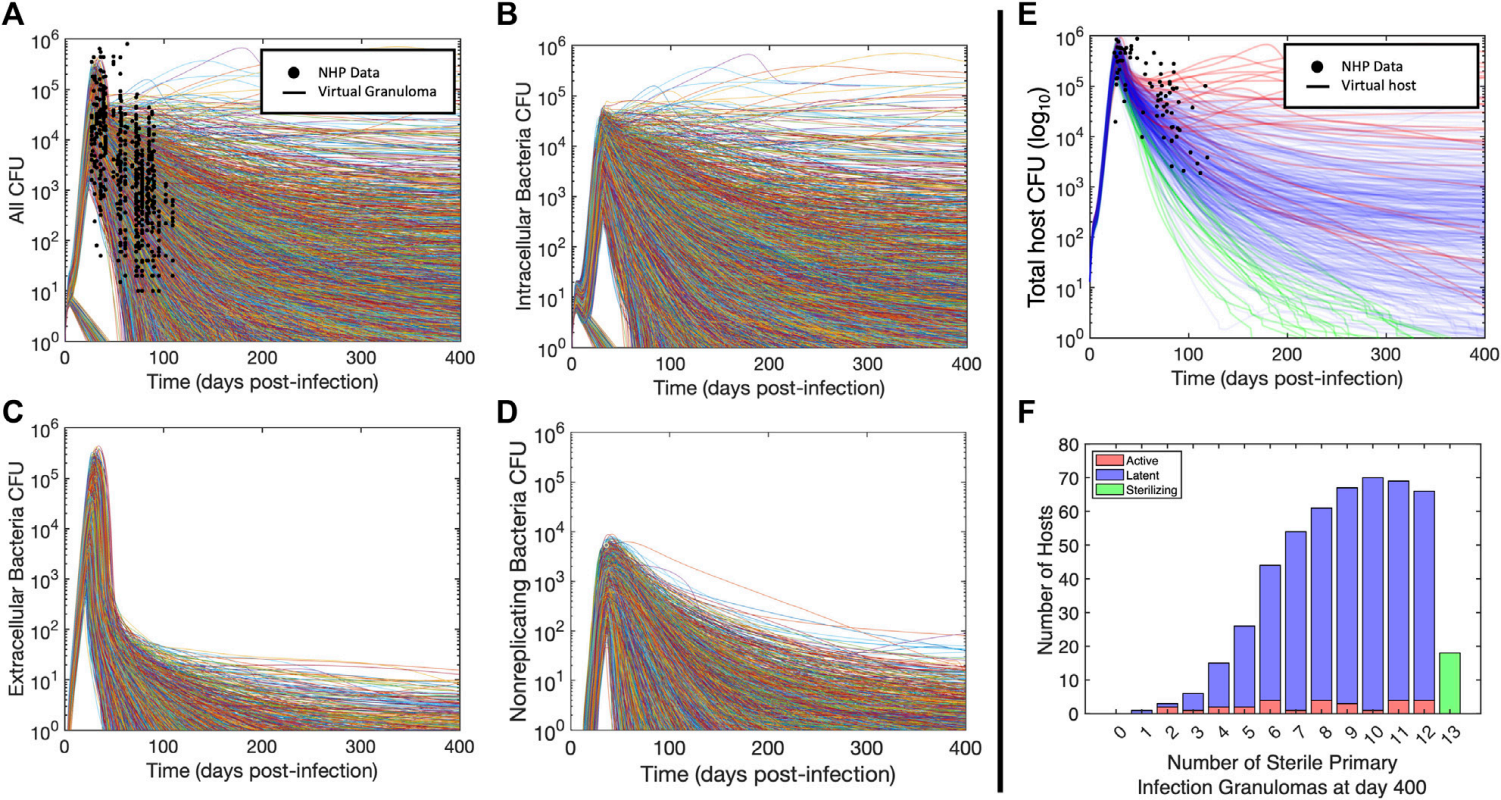
Virtual hosts and cohort for Mtb infection using *HostSim*. **(A–D)** Bacteria loads (CFU) for the total bacterial population and subpopulation trajectories for each granuloma. Curves showing granuloma CFU over time for each of the 13 primary granulomas in 500 hosts for 400 days post infection. Panel **(A)** shows total CFU per granuloma as well as the analogous measurements from NHPs at specific points (Gideon et al., 2015; Marino et al., 2016; Cadena et al., 2018; Darrah et al., 2019), **(B)** shows intracellular bacteria, **(C)** shows extracellular bacteria, and **(D)** shows nonreplicating bacteria. **(E)** Curves showing total lung CFU for each of 500 virtual hosts. Trajectories are colored by the virtual host classification as sterilizing, latent, or active. We have also show whole-lung CFU counts from published NHP studies (Gideon et al., 2015; Marino et al., 2016; Cadena et al., 2018; Darrah et al., 2019) by summing CFU across all lung granulomas. **(F)** Population scale histogram of the number of sterilizing granulomas per virtual host out of 500 virtual hosts.

We validated our virtual hosts at multiple scales according to the ten simple rules credibility standard (Erdemir et al., 2020; Fogarty et al., 2022; Nanda et al., 2023; Tatka et al., 2023). Figures 3A–E shows trajectories of 6,500 primary granulomas and whole-host CFU counts taken from 500 virtual hosts generated after we calibrated to multiple datasets from different NHP studies. At the population scale, clinically latent hosts had a range of 1–12 primary granulomas that eliminated all bacteria while hosts with active infection had 2–12 sterilized granulomas (Figure 3F). This recapitulates the common thinking that a single high-burden granuloma may determine the state of the Mtb host (i.e., active infection) (Lin et al., 2016). *HostSim* predicts that a small portion of granulomas are able to clear infection with innate response during early infection, which is presently not feasible to test *in vivo*. This is a feature of all of our computational and mathematical models of TB and it is believed to be a phenomenon that occurs in humans. On both the granuloma and host scale, we witness the presence of a transient high-CFU peak at approximately day 20, consistent with experimental observations (Gideon et al., 2015; Marino et al., 2016; Cadena et al., 2018; Darrah et al., 2019) (see Supplementary Material S1 Section 2 for details). Our updated *HostSim* model also is able to examine predictions that would match a PET-CT scan of a primate (human or NHP). We refer to this as FDG avidity, one of the only sources of time-series granuloma-scale data from live hosts and obtained via PET-CT scans (see Supplementary Material S1 Section 3 for details). FDG avidity is a measure of immune cell activity at the infection site within granulomas (Lin et al., 2013; Esmail et al., 2016). Supplementary Videos S1, S2 show the same representative latent virtual host developing granulomas over 400 days post-infection, with coloration based on their predicted FDG avidity values (comparable to NHP PET-CT images in Figure 1A of Ganchua et al. (Ganchua et al., 2018)).

### 3.3 Generating a virtual host VH at the whole-host scale and a virtual cohort $\left\{VH\right\}$ at the population scale

Our goal is to create a cohort of virtual hosts that mechanistically produce the trajectories of bacterial burden in time. We will use this virtual cohort to test interventions - either a treatment intervention, e.g., drugs, vaccines, etc. (or in some cases, a model modification). For an experimental treatment study, a cohort can be defined as both an infection population and an uninfected (negative) control population. Our “healthy” state is represented by steady-state levels of T-cells in the blood and LNs and resting macrophages within lungs (as we currently do not track host toxicity or tolerability in *HostSim*, we only use the infection model for drug studies). In our MID framework, we use a unique virtual population, our virtual cohort, on which we test our interventions to compare against the same virtual cohort against the untreated treatment control scenario $M_{0}$ . To that end, we want to have a virtual cohort whose members, (i.e., the virtual hosts) have a meaningful identity that can be interpreted independently from any specific model version.

We represent our virtual hosts, members of our virtual cohort $\left\{VH\right\}$ , by a collection of model parameters, $P_{i}$ chosen from a set of biologically valid ranges. We created a virtual cohort of 500 virtual hosts in this way by sampling from calibrated experimental ranges, as described in Section 2. Since our model versions share all parameter fields (except intervention parameters $A_{i}$ that do not vary between to virtual hosts), the same virtual host can be easily represented in either model version, known as the *virtual patient (host) identity*.

### 3.4 Drug interventions using *HostSim* ( $M_{P}$ )

A key aspect of creating a MID framework is to test interventions. For example, given the large drug regimen design space for diseases like TB, where multiple drugs are given for long periods of time, the possible combinations are on the order of 10^17^ (Cicchese et al., 2017)! The ability to explore the effects of drugs at the tissue, host, and population scales simultaneously in a virtual cohort is necessary to help screen this large space with the goal of identifying candidates that will be the best to test within a human cohort. A key goal of creating a MID framework is to use the impact of an intervention over multiple scales and to examine the wealth of synthetic data by comparing the outcomes of our virtual cohort with and without interventions.

To create an example intervention companion model $M_{P}$ to *HostSim,* we will define a single-drug-like intervention. We will assume that these drugs solely affect either bacterial replication and/or death rates depending on their known drug actions (Figure 2). INH, BDQ and PZA are three antibiotics that are commonly used to treat TB (Chahine et al., 2014; Prideaux et al., 2015; Nahid et al., 2016; Sarathy et al., 2016). INH and BDQ are known to have bactericidal activity, although BDQ is more efficient at killing Mtb within the necrotic caseum region of granulomas and can also be taken inside of infected macrophages. PZA is a bacteriostatic drug whose action we represent by halving the bacterial replication rate (see Section 2.5). Our simple representations of drug interventions here do not include consideration of pharmacokinetics, or the ability of drugs to penetrate well into granulomas as we have done previously (Pienaar et al., 2017; Budak et al., 2023). We define our impact quantifications in this setting to be a host impact score $H_{S}$ and a granuloma impact score $G_{S}$ . These are derived from CFU ratios between $M_{P}$ and $M_{0}$ , where zero-score is zero-efficacy, and positive scores indicate a beneficial intervention for virtual hosts (see Section 2.6). Importantly, the outcomes being measured are credible from both $M_{0}$ and $M_{P}$ respectively as i) mechanistically predicting CFU trajectories falls within their context of use, and ii) our goal for using our example $M_{P}$ to calculate $H_{s}$ and $G_{s}$ is to examine qualitative behavior of CFU reduction by Mtb subpopulation.

We described above how we generate our virtual cohort $\left\{VH\right\}$ . We then represent and simulate this virtual cohort in both the control version $M_{0}$ and drug intervention versions of *HostSim*: $M_{INH}$ , $M_{BDQ}$ , and $M_{PZA}$ , and we calculate the granuloma and host impact scores (see Section 2.6, expressions [1] and [2]). Together, these components give us a way to study the impact of interventions on our virtual cohort, allowing us to analyze intervention efficacy across the cell/tissue, whole-host, and population scales.

### 3.5 Granuloma and host scale analyses of drug intervention capture mechanistic insights

**FIGURE 4:**
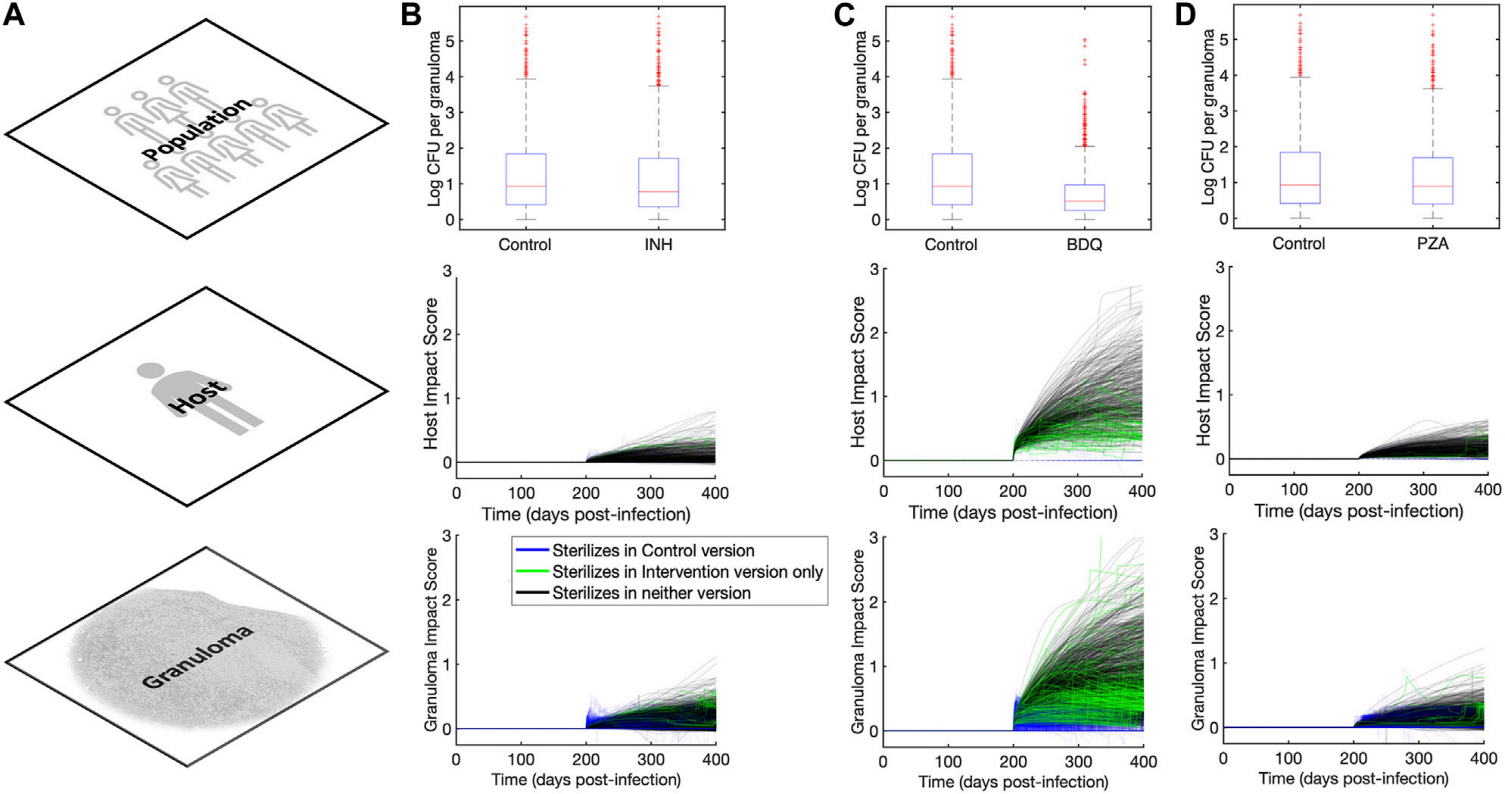
Impact quantification of three single-drug-like interventions across granuloma, host, and population scales. **(A)** Column showing the three scales (across rows) at which we analyze outcomes in our MID framework study. **(B–D)** Columns showing population, host, and granuloma scale impact quantification scores for **(B)** $M_{INH}$ , **(C)** $M_{BDQ}$ , and **(D)** $M_{PZA}$ versions of *HostSim*. Granuloma and host scale plots show the granuloma and host impact scores (Eqs 1, 2) across time for each granuloma and host, respectively. An impact score of 0 indicates equal CFU in $M_{0}$ and $M_{P}$ and higher impact scores indicate more favorable host outcomes. Blue lines show granuloma and host trajectories that are sterilized in the control group by day 400, green lines show granulomas and hosts that sterilized only in the intervention version, and black lines indicate trajectories that sterilized in neither control nor intervention cases. The population scale bar plots show a direct comparison between the control version and the intervention version at day 400, highlighting that the variation of the impact efficacy is obfuscated if individual host trajectories are not tracked.

As the final component of our MID framework, we want to understand how the perturbation or treatment $M_{P}$ affects our virtual cohort over multiple physiological scales. With our drug interventions defined above, we use the impact quantification method described in Section 2 to compare outcomes of granulomas and hosts in the non-treatment control scenario against the three drug treatment scenarios. Figure 4 shows the impact quantification of the 3 different drug interventions at all three physiological scales. We begin treatment at day 200 and treat for 200 days post-infection. At all three scales, the impact scores suggest that $M_{BDQ}$ is the most effective drug, which is consistent with how we defined it as compared to $M_{INH}$ . Interestingly, there is a wide range of impact scores on both the granuloma and host scales, even if statistics on CFU counts at the population scale would not directly reveal this (Figure 4; Table 2). In many granulomas, treatment did not help much - indicated by an impact score near 0. Many granulomas with low impact scores either cleared in both model versions or cleared in the $M_{P}$ version only (as a result of granulomas starting treatment with low CFU burden in the control case). However, we observed many granulomas with low impact scores (<0.5) that remained infected in both $M_{0}$ and $M_{P}$ , indicating that some granulomas resist treatment more than others. This may depend on the action of a drug, on the host immune response or on the bacterial levels at the start of treatment. The population scale comparison between the control and intervention cases suggests that bactericidal interventions (as in the case of $M_{BDQ}$ and $M_{INH}$ ) are a more effective action for a drug intervention (middle and bottom row panels). We observe, however, that the pooled cohort data (top row panels) cannot be used as accurately to predict whether or not a drug will help an individual host. This demonstrates the importance of developing a MID framework that captures both granuloma-scale and host-scale intervention responses that cannot be detected purely at a population level.

**TABLE 2:** Impact of interventions of three different drugs on a virtual cohort with 500 hosts across multiple scales.

Feature	$M_{0}$	$M_{INH}$	$M_{PZA}$	$M_{BDQ}$
Percentage of sterilizing hosts in population	3.6%	4.2%	5.0%	12.0%
Percentage of hosts with active infection in population	5.6%	4.4%	4.0%	3.8%
Hosts that reduced CFU by 200 days post-intervention	-	53%	91%	96%
Granulomas that reduced CFU by 200 days post-intervention	-	16%	26%	32%
Granulomas that sterilized	67%	68%	69%	77%

Another way to explore intervention impact scores is to understand variance of intervention efficacy. We analyzed host and granuloma impact scores as model outputs using a sensitivity analysis that considers non-linear correlations, called partial rank correlation (see Section 2.7). This method correlates non-linear model parameters to outputs of interest, and in this case, we can use both scale impact scores as a readout. The results shown in Table 3 suggest that many host model parameters impact the BDQ-like drug intervention. As BDQ is shown to have the largest possible intervention impact score of the three drugs that we studied (Figure 4; Table 2) as well as the widest variance of impact scores, we found it surprising that BDQ also interacts with the highest numbers of host parameters. It may be that interventions that interact with many model components may both reach higher efficacy but also have a more complex range of host responses. Moreover, we find that parameters that correlate with the impact of drug interventions also overlap with the parameters that impact FDG avidity outputs (i.e., a measure of host immune activity) (Supplementary Material S1 Section 3.2). What this tells us is that expressions FDG avidity, as predicted by expressions [S1-S2], is driven by the same parameters that drive our impact score. This may suggest that FDG avidity is a good predictor of projected intervention efficacy, or that both quantities are affected by the same mechanisms.

**TABLE 3:** Descriptions of parameters significantly driving variance in granuloma impact scores for three different treatments. PRCC values remained unchanged qualitatively between days 200 and 400 so, for simplicity, only the trends are shown. We use + to indicate a positive correlation after intervention, and - to indicate a negative correlation, and “n/a" indicates no significant correlation. Trends indicated correspond to PRCC values that were filtered by PCC z-test (Marino et al., 2008) to control for the absolute magnitude of the intervention impact.

Parameter description	Efficacy correlation with $M_{INH}$	Efficacy correlation with $M_{BDQ}$	Efficacy correlation with $M_{PZA}$
In-macrophage carrying capacity of Mtb	++	++	++
Resting macrophage infection rate constant	n/a	+	++
Half-saturation of Mtb in infected macrophages	n/a	-	-
Decay rate constant of Interleukin-10	n/a	-	n/a

## 4 Discussion

We introduce a model analysis framework that can be used to track a virtual cohort and the impacts of interventions or other model perturbations across multiple physiological scales patient, that we refer to as a MID framework. The three components of a MID framework are i) a cohort of a virtual patients (or virtual hosts) consistent across model versions; ii) validated control and intervention model versions; and iii) an explicitly defined method of impact quantification. A MID framework leverages the ability of models to perform “what-if” experiments on the same virtual patient under different interventions and is able to decompose the spectrum of patient responses to predict system parameters - and thereby also individual model components - as being principally responsible for patient placement within a spectrum.

As part of creating a MID framework, we developed an updated version of our whole-host model of TB, *HostSim*, which ranges from the cell/tissue scale to the population scale. We calibrated this model to both experimental data from the Flynn lab (Gideon et al., 2015; Marino et al., 2016; Cadena et al., 2018; Darrah et al., 2019), and to synthetic data from our fine-grained model *GranSim*, which is an agent-based model that represents formation and function of individual granulomas. TB is an ideal candidate for implementation of a MID framework as it is complex and intrinsically multi-scale, which necessarily requires many parameters. Moreover, model outcomes from *HostSim* (e.g., CFU count and FDG avidity) are directly comparable to existing data and can be used to create and interrogate intuitive impact quantification measures.

We presented an example MID framework implementation to generate examples of quantitative, mechanism-based outcome predictions for interventions that are challenging to obtain experimentally and may be used to forecast outcome heterogeneity for future experiments. We used our TB-focused MID framework to analyze the impact of three different drug interventions–each of which phenomenologically represents a drug commonly used to treat TB–on a virtual cohort of 500 virtual hosts. In doing so, we captured and quantified the impact of different interventions at multiple scales, which is typically inaccessible to an experimental-like research design that usually occurs over a single scale. Our method shows that the parameters - and thereby mechanisms - most correlated with host responsiveness to drugs overlap with the parameters most that correlate with our prediction a non-invasive, spatial measurement of TB infection progression, FDG avidity.

Though we use a MID framework to study virtual human patients in the context of virtual clinical trials, the method is not tied to this application. Given a model system, one may develop intervention model versions for other forms of interventions after you have a suitable control version–e.g., host-directed therapies, vaccines, or booster efficacies. Indeed, there are existing model studies that employ virtual-cohort-like methods of analyses. However, without specific attention paid to each of the three components of a MID framework, *ad hoc* approaches may face i) an ill-defined notion of a virtual patient (or subject), such that it is difficult to determine whether the “same subject” is being represented in both model versions; ii) non-rectifiable or non-credible model versions, where the control version $M_{0}$ and the intervention version $M_{P}$ are intractably different as in the case of a singular perturbation, and iii) an improperly constructed intervention quantification method which may bias or overly-abstract model outputs and thus preclude meaningful interpretation. Improper impact quantification selection may cause us to use model output outside of its context of use, and lead to subtly non-credible comparisons.

Another use of the MID framework may be to examine impacts of model updates, allowing us to demonstrate model consistency. If a model is updated extensively, we could use the original model as $M_{0}$ and the updated model as a new version $M_{0}^{\prime }$ instead of an $M_{P}$ . In this case, minimal deviations would suggest that very little changed by way of introducing the new components–perhaps ideal for surrogate modeling, or more informative about the impact of fine-graining a model (Kirschner et al., 2014). Any simplification or re-representation of a model subcomponent could be examined in this way if model outputs and classifications are able to be meaningfully compared.

There are other advantages to having a MID framework. First, a calibrated virtual cohort annotated with MID-framework outcomes may be used to store virtual reference cases. That is, for computationally intensive models, it may be useful to store virtual hosts across a heterogeneous virtual cohort along with their control ( $M_{0}$ ) and intervention ( $M_{P}$ ) outcomes for comparison to quantitatively-similar real hosts. If a clinical patient or an experimental subject can be measured in such a way that we can find their nearest *digital partners*, then pre-simulated fine-grained virtual patients may be used to approximate both their untreated and treated outcomes. In this way, we may quantitatively rank the most effective treatment for a given real host, scaled with some confidence measure representing the “closeness” of the clinical host to their nearest digital partner. If the model is not entirely identifiable given live patient data, this will yield a twofold benefit: 1) a *family of nearest digital partners* identified by what data is available together with a forecast cone, which quantifies how those partners diverge over time; and 2) a clear and immediate use for new, multi-modal data. Including new data will whittle down the family of digital partners and narrow the forecast cone. We may also use the digital partner framework with existing models to best identify what modes of new data will best improve patient forecasting and illuminate what types of data will best improve parameter identification. This is particularly important when using mechanistic models for generating synthetic data for other applications (An and Cockrell, 2023). Lastly, we can continue to add more and more virtual patients to virtual cohorts as needed: generating virtual patients around a given human subject whose nearest digital partner lies in a sparsely-sampled region of parameter space will allow us to dynamically populate the virtual patient cohort to the needs of the real patient population.

Related efforts have been made to create tools that leverage computational models for medical decision supplementation and research (Vodovotz and An, 2019; Foy et al., 2020; Joshi et al., 2020; Wright and Davidson, 2020; Laubenbacher et al., 2022; Venkatapurapu et al., 2022), or for autonomous medical decision-making (Hoffmann et al., 2020; Singh et al., 2022; Yang, 2022) as a form of personalized medicine. A digital twin is a tool that predicts future states within a specific, real, complex biomedical system using a flexible, calibrated multi-scale computational model that integrates available real-time host-specific data. Medical digital twins (MDTs) have been developed to replicate and predict the trajectory of specific patients’ diseases (Wright and Davidson, 2020; Masison et al., 2021; Laubenbacher et al., 2022). With recent demand for standardization of and development of MDT validation, uncertainty analysis, model linkage, and interpretable outcomes (Wright and Davidson, 2020; Laubenbacher et al., 2022), the ability to find digital partners within virtual cohorts created from digital twins and the associated response to treatment would be a powerful decision supplement tool.

It is worth noting the distinction between a MID framework and several related sensitivity analysis tools. Existing sensitivity analyses, including both local and global methods, uncover dependencies between a model’s input variables and outcomes. While these tools (such as PRCC, used in this paper) are extremely valuable, they often include comparison of many parameter values distributed through a range. Often in experiments, intervention methods are defined regimens - a procedure applied to all subjects of the study (e.g., having multiple patients test the same FDA-approved drug dosage). In these cases, it is preferable to have an in-depth look at the impact of a single intervention regimen on an individual, as opposed to sampling a “gradient of intervention magnitude” - e.g., testing with/without drug, as opposed to various dosages. This is also true in the case of MDTs: having more detailed information on the projected impact of two mechanistically distinct interventions on a single patient may be invaluable. Moreover, $M_{P}$ and $M_{0}$ may differ by more than a single parameter perturbation (e.g., a new cell type being considered in $M_{P}$ ). In these cases, comparison between $M_{0}$ and $M_{P}$ is substantially distinct from a local sensitivity analysis.

Importantly, using a MID framework is not a substitute for rigorous and validated model construction, nor do we wish anybody to consider our MID framework as such. Instead, it is a method to analyze differences between two highly-related, credible, multi-scale models by separating out those components that are patient-specific and those components that are intervention-specific. Each individual model version should be considered as a trial procedure - such as experimental or a placebo group protocol-that is being applied to the *same* virtual host. Each model version should be able to make credible claims about host outcomes in each intervention scenario; and the MID framework is a systematic method for examining drivers of heterogeneity of the response to those interventions.

In the future, our *HostSim*-derived virtual cohort may be improved by the use of experimental distributions for each parameter in the model, instead of uniformly sampling from each range. This would ensure that the virtual cohorts in our MID framework capture the demographic of host heterogeneity in more detail. This may grant us more insights both into subtle differences between common presentations of TB at each scale, or it may allow us to predict outlier or unusual host presentations or responses. It is also worth noting that the three steps of creating a MID framework, while conceptually simple, must be considered carefully. Creation of intervention models may be straightforward in some cases, but there should be a limiting case where the control case can be recovered by reducing the intervention’s amplitude. Representing a real-world entity (e.g., in the case of MDTs) in each model version may embed assumptions about that host that could be inconsistent between the model versions if each version’s assumptions are not stated explicitly. Finally, the intervention impact quantification method should be free of biases that might favor one phenotype as more easily impacted than another and should not overreach the context of use of either model version.

## Data Availability

The original contributions presented in the study are included in the article/Supplementary Material, further inquiries can be directed to the corresponding author.
