# Supplementary material for: A framework for multi-scale intervention modeling: virtual cohorts, virtual clinical trials, and model-to-model comparisons
Source: Front Syst Biol. 2024 Jan 22;3:1283341. doi: 10.3389/fsysb.2023.1283341 (PMC11415237; doi:10.3389/fsysb.2023.1283341)
Supplement: Supplementary file 1 [file DataSheet2.PDF]

SUPPLEMENTARY MATERIAL 2 - *HOSTSIM* TECHNICAL DESCRIPTION

This document contains the equations for each compartment in *HostSim*, how they are coupled, parameter ranges, and initial conditions. *HostSim* was originally published by Louis Joslyn, Jennifer Linderman, and Denise Kirschner in the article "A virtual host model of Mycobacterium tuberculosis infection identifies early immune events as predictive of infection outcomes" in 2022 in the Journal of Theoretical Biology.

We have separated this document into sections for readability. In section 1, we detail all of the equations in each granuloma compartment. In section 2, we detail the equations in the lymph node and blood compartments. In section 3, we present the method by which all of our compartments are coupled. In section 4, we describe how we model dissemination - that is, how we represent one granuloma causing another to form. In section 5, we present tables for the parameter ranges and initial condition values for our granuloma (section 5.1), lymph node and blood (subsection 5.2) equations. In each of these sections, we included both a symbolic variable for each variable (e.g., M_R or ξ_2) and a corresponding searchable variable (e.g., MR or xi2) for convenience of navigating this document.

1. Granuloma equations

Each granuloma will have its own instance of the equations presented in the subsections below. Each host has a "host-scale granuloma parameter base" selected by the LHS sampling scheme using the ranges presented in Section 5.1. To capture intra-host variability, individual granulomas within a host are given parameter values normally distributed around this base host value. Terms indicated by $Pulled\left[\cdot\right]_g$ involve quantities pulled from the lymph node and blood equations, and is described in Section 3. Below is an index of all state variables in granulomas.

State Variable	Searchable name	Units	Description
M_R	MR	count	Resting macrophages
M_I	MI	count	Infected macrophages
M_A	MA	count	Activated macrophages
B_I	BI	count	Intracellular Mtb
B_E	BE	count	Extracellular Mtb
B_N	BN	count	Non-replicating Mtb
Ca	CA	mass of $M\phi$	Necrotic tissue mass
T_0^4	T40	count	Mtb-specific Primed CD4$^+$ T-cells
T_1^4	Th1	count	Mtb-specific Th1 CD4$^+$ T-cells
T_2^4	Th2	count	Mtb-specific Th2 CD4$^+$ T-cells
T_{Non}^4	CD4Non	count	Nonspecific CD4$^+$ T-cells
T_{EM}^8	EMCD4	count	Mtb-specific CD4$^+$ effector memory T-cells
T_0^8	T80	count	Mtb-specific Primed CD8$^+$ T-cells
T_C	TC	count	Mtb-specific Cytotoxic CD8$^+$ T-cells
T^8	T8	count	Mtb-specific Effector CD8$^+$ T-cells
T_{EM}^8	EMCD8	count	Mtb-specific CD8$^+$ effector memory T-cells
T_{Non}^8	CD8Non	count	Nonspecific CD8$^+$ T-cells
F_α	TNF	pg/mL	TNF-α concentration
I_γ	IG	pg/mL	IFN-γ concentration
I_{12}	I12	pg/mL	Interleukin 12 concentration
I_{10}	I10	pg/mL	Interleukin 10 concentration
I_4	I4	pg/mL	Interleukin 4 concentration

1.1. Macrophage equations.

M_R - MR - Resting Macrophage count

$$\frac{d}{dt}(M_R) = \underbrace{\alpha_{4a}\left(M_A + w_2 M_I\right)}_{\text{Macrophage-driven recruitment}} \underbrace{+ Sr_{4b}\left(\frac{F_\alpha}{F_\alpha + f_8 I_{10} + s_{4b}}\right)}_{\text{TNF driven recruitment}} \underbrace{- k_2 M_R\left(\frac{B_E}{B_E + c_9}\right)}_{\text{Infection of } M_R}$$
$$\underbrace{- k_3 M_R\left(\frac{B_E + wB_I + \beta F_\alpha}{B_E + wB_I + \beta F_\alpha + c_8}\right)\left(\frac{I_\gamma}{I_\gamma + f_1 I_4 + f_7 I_{10} + s_1}\right)}_{\text{Activation of macrophages}} - \underbrace{\mu_{M_R} M_R}_{\text{Natural death}}$$

M_I - MI - Infected macrophages

$$\frac{d}{dt}(M_I) = \underbrace{k_2 M_R\left(\frac{B_E}{B_E+c_9}\right)}_{\text{Infection of } M_R} - \underbrace{k_{17} M_I\left(\frac{B_I^2}{B_I^2+(NM_I)^2}\right)}_{\text{Bursting of } M_I} - \underbrace{k_{14a} M_I\left(\frac{\left(\frac{T_C+w_3T_1^4}{M_I}\right)}{\left(\frac{T_C+w_3T_1^4}{M_I}\right)+c_4}\right)}_{\text{T-cell driven apoptosis of } M_I}$$
$$- \underbrace{k_{14b} M_I\left(\frac{F_\alpha}{F_\alpha+f_9I_{10}+s_{4b}}\right)}_{\text{TNF driven apoptosis of } M_I} - \underbrace{k_{52} M_I\left(\frac{\left(\frac{T_C\left(\frac{T_1^4}{T_1^4+C_{T_1^4}}\right)+w_1T_1^4}{M_I}\right)}{\left(\frac{T_C\left(\frac{T_1^4}{T_1^4+C_{T_1^4}}\right)+w_1T_1^4}{M_I}\right)+c_{52}}\right)}_{\text{Cytotoxic T-cell driven } M_I \text{ apoptosis}} - \underbrace{\mu_{M_I} M_I}_{\text{Natural death}}$$

M_A - MA - Activated macrophages

$$\frac{d}{dt}(M_A) = \underbrace{k_3 M_R\left(\frac{B_E+wB_I+\beta F_\alpha}{B_E+wB_I+\beta F_\alpha+c_8}\right)\left(\frac{I_\gamma}{I_\gamma+f_1I_4+f_7I_{10}+s_1}\right)}_{\text{Activation of resting macrophages}} - \underbrace{k_4 M_A\left(\frac{I_{10}}{I_{10}+s_8}\right)}_{\text{Deactivation by } I_{10}} - \underbrace{\mu_{M_A} M_A}_{\text{Natural death}}$$

1.2. **Mtb and caseum equations.**

B_I - BI - Intracellular bacterium count

$$\frac{d}{dt}(B_I) = \underbrace{\alpha_{19}\frac{B_I}{M_I}M_I\left(1-\frac{(B_I/M_I)}{N}\right)}_{\text{Replication}} + \underbrace{k_2\frac{N}{2}M_R\left(\frac{B_E}{B_E+c_9}\right)}_{\text{Internalization of } B_E} - \underbrace{k_{17}NM_I\left(\frac{B_I^2}{B_I^2-(NM_I)^2}\right)}_{\text{Bursting of } M_I}$$
$$- \underbrace{k_{14a}\frac{B_I}{M_I}M_I\left(\frac{\left(\frac{T_C+w_3T_1^4}{M_I}\right)}{\left(\frac{T_C+w_3T_1^4}{M_I}\right)+c_4}\right)}_{\text{T-cell driven } M_I \text{ apoptosis}} - \underbrace{k_{14b}\frac{B_I}{M_I}M_I\left(\frac{F_\alpha}{F_\alpha+f_9I_{10}+s_{4b}}\right)}_{\text{TNF driven aptosis}}$$
$$- \underbrace{k_{52}\frac{B_I}{M_I}M_I\left(\frac{\left(\frac{T_C\left(\frac{T_1^4}{T_1^4+C_{T_1^4}}\right)+w_1T_1^4}{M_I}\right)}{\left(\frac{T_C\left(\frac{T_1^4}{T_1^4+C_{T_1^4}}\right)+w_1T_1^4}{M_I}\right)+c_{52}}\right)}_{\text{Cytotoxic T-celldriven} M_I \text{ apoptosis}} - \underbrace{\mu_{B_I} B_I}_{\text{Natural Death}} - \underbrace{\mu_{M_I}\frac{B_I}{M_I}M_I}_{\text{Natural death of } M_I}$$

B_E - BE - Extacellular Bacterium Count

$$\frac{d}{dt}(B_E) = \underbrace{\alpha_{20} B_E \left(1 - \frac{B_E}{10^6}\right)}_{\text{Replication}} + \underbrace{(1 - C_N)}_{\text{Fraction outside of Caseum}} \Bigg[\underbrace{k_{17} N M_I \left(\frac{B_I^2}{B_I^2 + (N M_I)^2}\right)}_{\text{Macrophage bursting}} + \underbrace{k_{14a} N_{fracc} \frac{B_I}{M_I} M_I \left(\frac{\left(\frac{T_C + w_3 T_1^4}{M_I}\right)}{\left(\frac{T_C + w_3 T_1^4}{M_I}\right) + c_4} \right)}_{\text{T-cell driven apoptosis}}$$
$$+ \underbrace{k_{14b} N_{fraca} \frac{B_I}{M_I} M_I \left(\frac{F_\alpha}{F_\alpha + f_9 I_{10} + s_{4b}}\right)}_{\text{TNF driven apoptosis}} + \underbrace{\mu_{M_I} N_{fracd} \frac{B_I}{M_I} M_I}_{\text{Natural death of } M_I} \Bigg] + \underbrace{k_{Rev} N_{Ca} M_A Ca \cdot B_N}_{\text{Revealing of } B_N}$$
$$- \underbrace{k_2 \frac{N}{2} M_R \left(\frac{B_E}{B_E + c_9}\right)}_{\text{Internalization of } B_E} - \underbrace{k_{15} M_A B_E}_{M_A \text{ killing of } B_E} - \underbrace{k_{18} M_R B_E}_{M_R \text{ killing of } B_E} - \underbrace{\mu_{B_E} B_E}_{\text{Natural death}}$$

B_N - BN - Nonreplicating bacterium count

$$\frac{d}{dt}(B_N) = \underbrace{C_N}_{\text{Fraction inside of Caseum}} \Bigg\{ \underbrace{k_{17} M_I \left(\frac{B_I^2}{B_I^2 + (N M_I)^2}\right)}_{\text{Macrophage bursting}} + \underbrace{k_{14a} N_{fracc} \frac{B_I}{M_I} M_I \left(\frac{\left(\frac{T_C + w_3 T_1^4}{M_I}\right)}{\left(\frac{T_C + w_3 T_1^4}{M_I}\right) + c_4} \right)}_{\text{T-cell driven apoptosis}}$$
$$+ \underbrace{k_{14b} N_{fraca} \frac{B_I}{M_I} M_I \left(\frac{F_\alpha}{F_\alpha + f_9 I_{10} + s_{4b}}\right)}_{\text{TNF driven apoptosis}} + \underbrace{\mu_{M_I} N_{fracd} \frac{B_I}{M_I} M_I}_{\text{Natural death of } M_I} \Bigg\}$$
$$- \underbrace{\mu_{B_N} B_N}_{\text{Natural death}} - \underbrace{B_N k_{Rev} N_{Ca} Ca \cdot M_A}_{\text{Revealing of } B_N}$$

Ca - CA - Caseum

$$N_f \frac{d}{dt}(Ca) = \underbrace{k_{17} M_I \left(\frac{B_I^2}{B_I^2 + (N M_I)^2}\right)}_{\text{Bursting of } M_I} + \underbrace{k_{14a} M_I \left(\frac{\left(\frac{T_C + w_3 T_1}{M_I}\right)}{\left(\frac{T_C + w_3 T_1}{M_I}\right) + c_4} \right)}_{\text{T-cell mediated apoptosis of } M_I} + \underbrace{k_{14b} M_I \left(\frac{F_\alpha}{F_\alpha + f_9 I_{10} + s_{4b}}\right)}_{\text{TNF-mediated apoptosis of } M_I}$$
$$+ \underbrace{k_{52} \frac{B_I}{M_I} M_I \left(\frac{\left(\frac{T_C\left(\frac{T_1^4}{T_1^4 + C_{T_1^4}}\right) + w_1 T_1^4}{M_I}\right)}{\left(\frac{T_C\left(\frac{T_1^4}{T_1^4 + C_{T_1^4}}\right) + w_1 T_1^4}{M_I}\right) + c_{52}} \right)}_{\text{Cytotoxic T-cell mediated apoptosis of } M_I} - \underbrace{N_{Ca} Ca M_A}_{\text{Neutrophil-driven caseum clearance}}$$
$$+ \underbrace{\mu_{M_I} M_I + \mu_{M_A} M_A + \mu_{M_R} M_R \left(\frac{F_\alpha}{F_\alpha + f_9 I_{10} + s_{4b}}\right)}_{\text{Natural death of all macrophage populations}}$$

1.3. **CD4$^+$ T-cells.**

T_0^4 - T40 - Primed CD4$^+$ T-Cell count

$$\frac{d}{dt}\left(T_0^4\right) = Pulled\left[T_0^4\right]_g + \underbrace{\alpha_2 T_0^4\left(\frac{M_A}{M_A+c_{15}}\right)}_{\text{Proliferation}} - \underbrace{k_6 I_{12} T_0^4\left(\frac{I_\gamma}{I_\gamma+f_1 I_4+f_7 I_{10}+s_1}\right)}_{\text{Differentiation to } T_1^4}$$
$$- \underbrace{k_7 T_0^4\left(\frac{I_4}{I_4+f_2 I_\gamma+s_2}\right)}_{\text{Differentiation to } T_2^4} - \underbrace{\mu_{T_0} T_0}_{\text{Natural death}}$$

T_1^4 - Th1 - Effector CD4$^+$ T-cell count

$$\frac{d}{dt}\left(T_1^4\right) = \underbrace{k_6 I_{12} T_0^4\left(\frac{I_\gamma}{I_\gamma+f_1 I_4+f_7 I_{10}+s_1}\right)}_{\text{Differentiation from } T_0^4} + \underbrace{k_{31} T_{EM}^4 M_I\left(\frac{M_I}{M_I+3}\right)}_{\text{Differentiation of } T_{EM}^4}$$
$$- \underbrace{\mu_{T_\gamma}\left(\frac{I_\gamma}{I_\gamma+c}\right) T_1^4 M_A}_{\text{IFN}\gamma \text{ apoptosis of } T_0^1} - \underbrace{\mu_{T_1^4} T_1^4}_{\text{Natural death}}$$

T_2^4 - Th2 - Effector Th2 CD4$^+$ T-cell count

$$\frac{d}{dt}\left(T_2^4\right) = \underbrace{k_7 T_0^4\left(\frac{I_4}{I_4+f_2 I_\gamma+s_2}\right)}_{\text{Differentiation from } T_0^4} + \underbrace{k_{32} T_{EM}^4 M_A}_{\text{Differentiation from } T_{EM}^4} - \underbrace{\mu_{T_2^4} T_2^4}_{\text{Natural death}}$$

T_{EM}^4 - EMCD4 - Effector memory CD4$^+$ T-cell count:

$$\frac{d}{dt}\left(T_{EM}^4\right) = Pulled\left[T_{EM}^4\right]_g - \underbrace{k_{31} T_{EM}^4 M_I\left(\frac{M_I}{M_I+3}\right)}_{\text{Differentiation to } T_1^4} - \underbrace{k_{32} T_{EM}^4 M_A}_{\text{Differentiation to } T_2^4} - \underbrace{\mu_{T_{EM}^4} T_{EM}^4}_{\text{Natural death}}$$

T_{Non}^4 - CD4Non - Non-cognate CD4$^+$ T-cell count:

$$\frac{d}{dt}\left(T_{Non}^4\right) = Pulled\left[T_{Non}^4\right]_g - \underbrace{\mu_{T_{Non}^4} T_{Non}^4}_{\text{Natural death}}$$

1.4. **CD8$^+$ T-cells.**

T_0^8 - T80 - Primed CD8$^+$ T-cell count:

$$\frac{d}{dt}\left(T_0^8\right) = Pulled\left[T_0^8\right]_g + \underbrace{\alpha_2 T_0^8\left(\frac{M_A}{M_A+c_{15}}\right)}_{\text{Proliforation}}$$
$$- \underbrace{k_6 I_{12} T_0^8\left(\frac{I_\gamma}{I_\gamma+f_1 I_4+f_7 I_{10}+s_1}\right)}_{\text{Differentiation to } T^8 \text{ and } T_C} - \underbrace{\mu_{T_0} T_0^8}_{\text{Natural death}}$$

T^8 - T8 - Effector CD8$^+$ T-cell count

$$\frac{d}{dt}\left(T^8\right) = \underbrace{(m) k_6 I_{12} T_0^8\left(\frac{I_\gamma}{I_\gamma+f_1 I_4+f_7 I_{10}+s_1}\right)}_{\text{Differentiation from } T_0^8} + \underbrace{k_{34} T_{EM}^8 M_I\left(\frac{M_I}{M_I+3}\right)}_{\text{Differentiation from } T_{EM}^8} - \underbrace{\mu_{T_{C\gamma}}\left(\frac{I_\gamma}{I_\gamma+c_c}\right) T^8 M_A}_{\text{IFN}\gamma \text{ apoptosis}} - \underbrace{\mu_{T_C} T_8}_{\text{Natural death}}$$

T_C - TC - Cytotoxic CD8$^+$ T-cell count

$$\frac{d}{dt}(T_C) = \underbrace{(1-m)k_6 I_{12} T_0^8 \left(\frac{I_\gamma}{I_\gamma + f_1 I_4 + f_7 I_{10} + s_1}\right)}_{\text{Differentiation from } T_0^8} + \underbrace{k_{33} T_{EM}^8 M_I \left(\frac{M_I}{M_I + 3}\right)}_{\text{Differentiation from } T_{EM}^8} - \underbrace{\mu_{T_{C\gamma}} \left(\frac{I_\gamma}{I_\gamma + c_c}\right) T_C M_A}_{\text{IFN}\gamma \text{ apoptosis}} + \underbrace{\mu_{T_C} T_C}_{\text{Natural death}}$$

T_{EM}^8- EMCD8 - Effector Memory CD8$^+$ T-cell count

$$\frac{d}{dt}\left(T_{EM}^8\right) = Pulled\left[T_{EM}^8\right]_g - \underbrace{k_{33} T_{EM}^8 M_I \left(\frac{M_I}{M_I+3}\right)}_{\text{Differentiation to } T_C} - \underbrace{k_{34} T_{EM}^8 M_I \left(\frac{M_I}{M_I+3}\right)}_{\text{Differentiation to } T^8} - \underbrace{\mu_{T_{EM}^8} T_{EM}^8}_{\text{Natural death}}$$

T_{Non}^8 -CD8Non - Non-cognate CD8$^+$ T-cell count

$$\frac{d}{dt}\left(T_{Non}^8\right) = Pulled\left[T_{Non}^8\right]_g - \underbrace{\mu_{T_{Non}^8} T_{Non}^8}_{\text{Natural death}}$$

1.5. **Cytokines.**

F_α - TNF -TNF-α concentration

$$\frac{d}{dt}(F_\alpha) = \underbrace{\alpha_{30} M_I}_{\text{Production by } M_I} + \underbrace{\alpha_{31} M_A \left(\frac{I_\gamma + \beta_2 (B_E + wB_I)}{I_\gamma + \beta_2 (B_E + wB_I) + f_1 I_4 + f_7 I_{10} + s_{10}}\right)}_{\text{IFN-IL mediated production of TNF}\alpha \text{ by } M_A}$$
$$+ \underbrace{\alpha_{32} T_1^4}_{\text{Production by } T_1^4} + \underbrace{\alpha_{33}\left(\frac{T_C + T^8}{2m}\right)}_{\text{Production by CD8}^+ \text{ T-cells}} - \underbrace{\mu_{F_\alpha} F_\alpha}_{\text{Decay}}$$

I_γ - IG - Interferon-gamma

$$\frac{d}{dt}(I_\gamma) = \underbrace{s_g \left(\frac{B_E + wB_I}{B_E + wB_I + c_{10}}\right)\left(\frac{I_{12}}{I_{12} + s_7}\right)}_{\text{Other}} + \underbrace{\alpha_{5a} T_1^4 \left(\frac{M_A}{M_A + c_{5a}}\right)}_{\text{Production by } T_1^4} + \underbrace{\alpha_{5b} T^8 \left(\frac{M_A}{M_A + c_{5b}}\right)}_{\text{Production by } T^8} + \underbrace{\alpha_{5c} M_I}_{\text{Production by } M_I}$$
$$+ \underbrace{\alpha_7 T_0^4 \left(\frac{I_{12}}{I_{12} + f_4 I_{10} + s_4}\right) + \alpha_7 T_0^8 \left(\frac{I_{12}}{I_{12} + f_4 I_{10} + s_4}\right)}_{\text{Interleukin-mediated production by primed T-cells}} - \underbrace{\mu_{I_\gamma} I_\gamma}_{\text{Decay}}$$

I_{12} - I12 - Interleukin-12

$$\frac{d}{dt}(I_{12}) = \underbrace{s_{12}\left(\frac{B_E + wB_I}{B_E + wB_I + c_{230}}\right)}_{\text{Other}} + \underbrace{\alpha_{23} M_R \left(\frac{B_E + wB_I}{B_E + wB_I + c_{23}}\right)}_{\text{Production by } M_R} + \underbrace{\alpha_8 M_A \left(\frac{s}{s + I_{10}}\right)}_{\text{Production by } M_A} - \underbrace{\mu_{I_{12}} I_{12}}_{\text{Decay}}$$

I_{10} - I10 - Interleukin-10

$$\frac{d}{dt}(I_{10}) = \underbrace{\delta_7 (M_I + M_A)\left(\frac{s_6}{I_{10} + f_6 I_\gamma + s_6}\right)}_{\text{IFN-IL mediated production by macrophages}} + \underbrace{\alpha_{16} T_1^4 + \alpha_{17} T_2^4}_{\text{Production by CD4}^+ \text{ T-cells}} + \underbrace{\alpha_{18}\left(\frac{T_C + T^8}{2m}\right)}_{\text{Production by CD8}^+ \text{ T-cells}} - \underbrace{\mu_{I_{10}} I_{10}}_{\text{Decay}}$$

I_4 - I4 - Interleukin-4

$$\frac{d}{dt}(I_4) = \underbrace{\alpha_{11} T_0^4}_{\text{Production by } T_0^4} + \underbrace{\alpha_{12} T_2^4}_{\text{Production by } T_2^4} - \underbrace{\mu_{I_4} I_4}_{\text{Decay}}$$

2. Lymph node and blood equations

The lymph node compartment will clonally expand and differentiate effector cells to be sent to the blood. Cells from the blood can be recruited to granulomas based on their request. We select each parameter in the following equations using the LHS sampling scheme using the ranges presented in Section 5.2. Terms indicated by $Pulled\left[\cdot\right]_g$ involve quantities pulled at each time step by lung granulomas, is described in Section 3. Below is an index of all state variables in the lymph nodes and blood.

Variable	Searchable name	Unit	Variable description
	**Lymph Node**		
APC	APC	count	Antigen-presenting cells in LN
N_4^{LN}	LnN4	count	Mtb-specific naive CD4$^+$ T-cell count in LN
P_4^{LN}	LnP4	count	Mtb-specific precursor CD4$^+$ T-cell count in LN
E_4^{LN}	LnE4	count	Mtb-specific effector CD4$^+$ T-cell count in LN
CM_4^{LN}	LnCM4	count	Mtb-specific central memory CD4$^+$ T-cell count in LN
EM_4^{LN}	LnEM4	count	Mtb-specific effector memory CD4$^+$ T-cell count in LN
$N_{Non,4}^{LN}$	LnN4Non	count	Nonspecific naive CD4$^+$ T-cell count in LN
$CM_{Non,4}^{LN}$	LnCM4Non	count	Nonspecific central memory CD4$^+$ T-cell count in LN
N_8^{LN}	LnN8	count	Mtb-specific naive CD8$^+$ T-cell count in LN
P_8^{LN}	LnP8	count	Mtb-specific precursor CD8$^+$ T-cell count in LN
E_8^{LN}	LnE8	count	Mtb-specific effector CD8$^+$ T-cell count in LN
CM_8^{LN}	LnCM8	count	Mtb-specific central memory CD8$^+$ T-cell count in LN
EM_8^{LN}	LnEM8	count	Mtb-specific effector memory CD8$^+$ T-cell count in LN
$N_{Non,8}^{LN}$	LnN8Non	count	Nonspecific naive CD8$^+$ T-cell count in LN
$CM_{Non,8}^{LN}$	LnCM8Non	count	Nonspecific central memory CD8$^+$ T-cell count in LN
	**Blood**		
N_4^B	BlN4	pg/mL	Mtb-specific naive CD4$^+$ T-cell count in blood
E_4^B	BlE4	pg/mL	Mtb-specific effector CD4$^+$ T-cell count in blood
CM_4^B	BlCM4	pg/mL	Mtb-specific central memory CD4$^+$ T-cell count in blood
EM_4^B	BlEM4	pg/mL	Mtb-specific effector memory CD4$^+$ T-cell count in blood
$N_{Non,4}^B$	BlN4Non	pg/mL	Nonspecific naive CD4$^+$ T-cell count in blood
$E_{Non,4}^B$	BlE4Non	pg/mL	Nonspecific effector CD4$^+$ T-cell count in blood
$CM_{Non,4}^B$	BlCM4Non	pg/mL	Nonspecific central memory CD4$^+$ T-cell count in blood
$EM_{Non,4}^B$	BlEM4Non	pg/mL	Nonspecific effector memory CD4$^+$ T-cell count in blood
N_8^B	BlN8	pg/mL	Mtb-specific naive CD8$^+$ T-cell count in blood
E_8^B	BlE8	pg/mL	Mtb-specific effector CD8$^+$ T-cell count in blood
CM_8^B	BlCM8	pg/mL	Mtb-specific central memory CD8$^+$ T-cell count in blood
EM_8^B	BlEM8	pg/mL	Mtb-specific effector memory CD8$^+$ T-cell count in blood
$N_{Non,8}^B$	BlN8Non	pg/mL	Nonspecific naive CD8$^+$ T-cell count in blood
$E_{Non,8}^B$	BlE8Non	pg/mL	Nonspecific effector CD8$^+$ T-cell count in blood
$CM_{Non,8}^B$	BlCM8Non	pg/mL	Nonspecific central memory CD8$^+$ T-cell count in blood
$EM_{Non,8}^B$	BlEM8Non	pg/mL	Nonspecific effector memory CD8$^+$ T-cell count in blood

2.1. Antigen Presenting Cells.

APC - APC - Antigen presenting cells. Received APCs are given from granulomas, which is detailed in Section 3.

$$\frac{d}{dt}\left(APC\right) = -\mu_5 APC + \sum_{i\in\{\text{Granulomas}\}} \text{Received from Granuloma } i$$

2.2. CD4$^+$ T-cells in lymph nodes.

N_4^{LN} - LnN4 - CD4$^+$ Mtb-specific Naive T-cell count (Lymph node)

$$\frac{d}{dt}\left(N_4^{LN}\right) = \alpha\left[\underbrace{k_1 N_4^B\left(\frac{APC}{APC+hs_1}\right)}_{\text{Cytokine-driveen recruitment}} + \underbrace{\xi_1 N_4^B}_{\text{LN Influx}}\right] - \underbrace{\xi_2 N_4^{LN}}_{\text{LN Efflux}} - \underbrace{k_2 N_4^{LN} APC}_{\text{Differentiation into } P_4^{LN}}$$

P_4^{LN} - LnP4 - CD4$^+$ Mtb-specific Precursor T-cell count (Lymph node)

$$\begin{aligned}\frac{d}{dt}\left(P_4^{LN}\right) = & \underbrace{k_2 N_4^{LN} APC}_{\text{Differentiation from } N_4^{LN}} + \underbrace{k_3 CM_4^{LN} APC}_{\text{Differentiation from } CM_4^{LN}} + \underbrace{k_4 P_4^{LN}\left(1-\frac{P_4^{LN}}{\rho_1}\right)\left(\frac{APC}{APC+hs_4}\right)}_{\text{Proliferation}} \\ & - \underbrace{k_5 P_4^{LN}\left(\frac{APC}{APC+hs_5}\right)}_{\text{Differention into } E_4^{LN}} - \underbrace{k_6 P_4^{LN}\left(1-\frac{APC}{APC+hs_5}\right)}_{\text{Differentiation into } CM_4^{LN}} - \underbrace{\mu_6 P_4^{LN}}_{\text{Natural death}}\end{aligned}$$

E_4^{LN}- LnE4 - CD4$^+$ Mtb-specific effector T-cell count (Lymph Node)

$$\frac{d}{dt}\left(E_4^{LN}\right) = \underbrace{k_5 P_4^{LN}\left(\frac{APC}{APC+hs_5}\right)}_{\text{Differention from } P_4^{LN}} - \underbrace{\xi_3 E_4^{LN}}_{\text{LN Efflux}} - \underbrace{k_7 E_4^{LN}}_{\text{Differentiation into } EM_4^{LN}}$$

CM_4^{LN}- LnCM4 - CD4$^+$ Mtb-specific central memory T-cell count (Lymph Node)

$$\begin{aligned}\frac{d}{dt}\left(CM_4^{LN}\right) = & \alpha\left[\underbrace{k_8 CM_4^B\left(\frac{APC}{APC+hs_8}\right)}_{\text{Cytokine-mediated recruitment}} + \underbrace{\xi_4 CM_4^B}_{\text{LN Influx}}\right] + \underbrace{k_6 P_4^{LN}\left(1-\frac{APC}{APC+hs_5}\right)}_{\text{Differentiation from } P_4^{LN}} \\ & - \underbrace{k_3 CM_4^{LN} APC}_{\text{Differentiation to } P_4^{LN}} - \underbrace{\xi_5 CM_4^{LN}}_{\text{LN Efflux}}\end{aligned}$$

EM_4^{LN} - LnEM4 - CD4$^+$ Mtb-specific effector memory T-cell count (Lymph Node)

$$\frac{d}{dt}\left(EM_4^{LN}\right) = \underbrace{k_7 E_4^{LN}}_{\text{Differentiation from } E_4^{LN}} - \underbrace{\xi_6 EM_4^{LN}}_{\text{LN Efflux}}$$

$N_{Non,4}^{LN}$ - LnN4Non - Non-cognate naive CD4$^+$ T-cell count

$$\frac{d}{dt}\left(N_{Non,4}^{LN}\right) = \alpha\left(\underbrace{k_1 N_{Non,4}^B\left(\frac{APC}{APC+hs_1}\right)}_{\text{Cytokine-mediated recruitment}} + \underbrace{\xi_1 N_{Non,4}^B}_{\text{LN Influx}}\right) - \underbrace{\xi_2 N_{Non,4}^{LN}}_{\text{LN Efflux}}$$

$CM_{Non,4}^{LN}$ - LnCM4Non - Non-cognate central memory CD4$^+$ T-cell count

$$\frac{d}{dt}\left(CM^{LN}_{Non,4}\right) = \alpha\left(\underbrace{k_8 CM^{B}_{Non,4}\left(\frac{APC}{APC+hs_8}\right)}_{\text{Cytokine-mediated recruitment}} + \underbrace{\xi_4 CM^{B}_{Non,4}}_{\text{LN Influx}}\right) - \underbrace{\xi_5 CM^{LN}_{Non,4}}_{\text{LN Efflux}}$$

2.3. CD8$^+$ T-cells in lymph nodes.

N_8^{LN} - LnN8 - Mtb-specific naive CD8$^+$ T-cell count

$$\begin{aligned}\frac{d}{dt}\left(N_8^{LN}\right) =& \alpha\left(\underbrace{k_{10} N_8^{B}\left(\frac{APC}{APC+hs_{10}}\right)}_{\text{Cytokine-mediated recruitment}} + \underbrace{\xi_7 N_8^{B}}_{\text{LN Influx}}\right) - \underbrace{\xi_8 N_8^{LN}}_{\text{LN Efflux}} \\ & - \underbrace{k_{11} N_8^{LN} APC\left(\frac{E_4^{LN} + w_{P_4} P_4^{LN}}{E_4^{LN} + w_{P_4} P_4^{LN} + hs_{11}}\right)}_{\text{Priming mediated by CD4-related cytokines}}\end{aligned}$$

P_8^{LN} - LnP8 - Mtb-specific precursor CD8$^+$ T-cell count

$$\begin{aligned}\frac{d}{dt}\left(P_8^{LN}\right) =& \underbrace{k_{11} N_8^{LN} APC\left(\frac{E_4^{LN} + w_{P_4} P_4^{LN}}{E_4^{LN} + w_{P_4} P_4^{LN} + hs_{11}}\right)}_{\text{Priming mediated by CD4-related cytokines}} + \underbrace{k_{12} CM_8^{LN} APC}_{\text{Differentiation from } CM_8^{LN}} \\ & + \underbrace{k_{13} P_8^{LN}\left(1 - \frac{P_8^{LN}}{\rho_1}\right)\left(\frac{APC}{APC+hs_{13}}\right)}_{\text{Cytokine-mediated proliferation}} - \underbrace{k_{15} P_8^{LN}\left(1 - \left\{\frac{APC}{APC+hs_{14}}\right\}\right)}_{\text{Differentiation to } CM_8^{LN}} \\ & - \underbrace{k_{14} P_8^{LN}\left\{\frac{APC}{APC+hs_{14}}\right\}}_{\text{Differentiation to } E_8^{LN}} - \underbrace{\mu_7 P_8^{LN}}_{\text{Natural death}}\end{aligned}$$

E_8^{LN} - LnE8 - Mtb-specific effector CD8$^+$ T-cell count

$$\frac{d}{dt}\left(E_8^{LN}\right) = \underbrace{k_{14} P_8^{LN}\left\{\frac{APC}{APC+hs_{14}}\right\}}_{\text{Differentiation from } P_8^{LN}} - \underbrace{\xi_9 E_8^{LN}}_{\text{LN Efflux}} - \underbrace{k_{16} E_8^{LN}}_{\text{Differentiation to } EM_8^{LN}}$$

CM_8^{LN} - LnCM8 - Mtb-specific central memory CD8$^+$ T-cell count

$$\begin{aligned}\frac{d}{dt}\left(CM_8^{LN}\right) =& \alpha\left[\underbrace{k_{17} CM_8^{B}\left(\frac{APC}{APC+hs_{17}}\right)}_{\text{Cytokine-mediated recruitment}} + \underbrace{\xi_{10} CM_8^{B}}_{\text{LN Influx}}\right] + \underbrace{k_{15} P_8^{LN}\left(1 - \left\{\frac{APC}{APC+hs_{14}}\right\}\right)}_{\text{Differentiation from } P_8^{LN}} \\ & - \underbrace{k_{12} CM_8^{LN} APC}_{\text{Cytokine-mediated differentiation to } P_8^{LN}} - \underbrace{\xi_{11} CM_8^{LN}}_{\text{LN Efflux}}\end{aligned}$$

EM_8^{LN} - LnEM8 - Mtb-specific effector memory CD8$^+$ T-cell count

$$\frac{d}{dt}\left(EM_8^{LN}\right) = \underbrace{k_{16} E_8^{LN}}_{\text{Differentiation from } E_8^{LN}} - \underbrace{\xi_{12} EM_8^{LN}}_{\text{LN Efflux}}$$

$N^{LN}_{Non,8}$ - LnN8Non - Non-cognate Naive CD8$^+$ T-cell count

$$\frac{d}{dt}\left(N_{Non,8}^{LN}\right)=\alpha\left[\underbrace{k_{10}N_{Non,8}^{B}\left(\frac{APC}{APC+hs_{10}}\right)}_{\text{Cytokine-mediated recruitment}}+\underbrace{\xi_7 N_{Non,8}^{B}}_{\text{LN Influx}}\right]-\underbrace{\xi_8 N_{Non,8}^{LN}}_{\text{LN Efflux}}$$

$CM_{Non,8}^{LN}$ - LnCM8Non - Non-cognate central memory CD8$^+$ T-cell count

$$\frac{d}{dt}\left(C_{Non,8}^{LN}\right)=\alpha\left[\underbrace{k_{17}CM_{Non,8}^{B}\left(\frac{APC}{APC+hs_{17}}\right)}_{\text{Cytokine-mediated recruitment}}+\underbrace{\xi_{10} CM_{Non,8}^{B}}_{\text{LN Influx}}\right]-\underbrace{\xi_{11} CM_{Non,8}^{LN}}_{\text{LN Efflux}}$$

2.4. CD4$^+$ T-cells in blood.

N_4^B - BlN4 - Mtb-specific naive CD4$^+$ T-cell count

$$\frac{d}{dt}\left(N_4^B\right)=\underbrace{\lambda S_{N_4}}_{\text{Source from Thymus}}+\underbrace{\alpha^{-1}\xi_2 N_4^{LN}}_{\text{LN Efflux}}-\underbrace{k_1 N_4^B\left(\frac{APC}{APC+hs_1}\right)}_{\text{Cytokine-mediated recruitment}}-\underbrace{\xi_1 N_4^B}_{\text{LN Influx}}-\underbrace{\mu_8 N_4^B}_{\text{Natural dath}}$$

E_4^B - BlE4 - Mtb-specific effector CD4$^+$ T-cell count

$$\frac{d}{dt}\left(E_4^B\right)=\underbrace{\alpha^{-1}\xi_3 E_4^{LN}}_{\text{LN Efflux}}-\underbrace{\mu_1 E_4^B}_{\text{Natural death}}-\sum_{g\in Granulomas} Pulled\left[E_4^B\right]_g$$

CM_4^B - BlCM4 - Mtb-specific central memory CD4$^+$ T-cell count

$$\frac{d}{dt}\left(CM_4^B\right)=\underbrace{\alpha^{-1}\xi_5 CM_4^{LN}}_{\text{LN Efflux}}-\underbrace{\xi_4 CM_4^B}_{\text{LN Influx}}-\underbrace{k_8 CM_4^B\left(\frac{APC}{APC+hs_8}\right)}_{\text{Cytokine-mediated recruitment}}$$

EM_4^B - BlEM4 - Mtb-specific effector memory CD4$^+$ T-cell count

$$\frac{d}{dt}\left(EM_4^B\right)=\underbrace{\alpha^{-1}\xi_6 EM_4^{LN}}_{\text{LN Efflux}}-\underbrace{\mu_2 EM_4^B}_{\text{Natural death}}-\sum_{g\in Granulomas} Pulled\left[EM_4^B\right]_g$$

$N_{Non,4}^B$ - BlN4Non - Non-cognate naive CD4$^+$ T-cell count

$$\frac{d}{dt}\left(N_{Non,4}^B\right)=\underbrace{(1-\lambda)\,s_{N_4}}_{\text{Production in thymus}}+\underbrace{\alpha^{-1}\xi_2 N_{Non,4}^{LN}}_{\text{LN Efflux}}-\underbrace{k_1 N_{Non,4}^B\left(\frac{APC}{APC+hs_1}\right)}_{\text{Cytokine-mediated recruitment}}-\underbrace{\xi_1 N_{Non,4}^B}_{\text{LN Influx}}-\underbrace{\mu_8 N_{Non,4}^B}_{\text{Natural death}}$$

$E_{Non,4}^B$ - BlE4Non - Non-cognate effector CD4$^+$ T-cell count

$$\frac{d}{dt}\left(E_{Non,4}^B\right)=\underbrace{s_{E_{Non,4}}}_{\text{Production in thymus}}-\underbrace{\mu_4 E_{Non,4}^B}_{\text{Natural death}}-\sum_{g\in Granulomas} Pulled\left[E_{Non,4}^B\right]_g$$

$CM_{Non,4}^B$ - BlCM4Non - Non-cognate central memory CD4$^+$ T-cell count

$$\frac{d}{dt}\left(CM_{Non,4}^B\right)=\underbrace{\alpha^{-1}\xi_5 CM_{Non,4}^{LN}}_{\text{LN Efflux}}-\underbrace{\xi_4 CM_{Non,4}^B}_{\text{LN Influx}}-\underbrace{k_8 CM_{Non,4}^B\left(\frac{APC}{APC+hs_8}\right)}_{\text{Cytokine-mediated recruitment}}$$

$EM_{Non,4}^B$ - BlEM4Non - Non-cognate effector memory CD4$^+$ T-cell count

$$\frac{d}{dt}\left(EM_{Non,4}^B\right)=\underbrace{s_{EM_{Non,4}}}_{\text{Source from thymus}}-\underbrace{\mu_2 EM_{Non,4}^B}_{\text{Natural death}}-\sum_{g\in Granulomas} Pulled\left[EM_{Non,4}^B\right]_g$$

2.5. **CD8$^+$ T-cells in blood.**

N_8^B - BlN8 Mtb-specific naive CD8$^+$ T-cell count

$$\frac{d}{dt}\left(N_8^B\right) = \underbrace{\lambda s_{N_8}}_{\text{Source from thymus}} + \underbrace{\alpha^{-1}\xi_8 N_8^{LN}}_{\text{LN efflux}} - \underbrace{k_{10} N_8^B \left(\frac{APC}{APC + hs_{10}}\right)}_{\text{Cytokine-mediated recruitment}} - \underbrace{\xi_7 N_8^B}_{\text{LN Influx}} - \underbrace{\mu_9 N_8^B}_{\text{Natural death}}$$

E_8^B - BlE8 - Mtb-specific effector CD8$^+$ T-cell count

$$\frac{d}{dt}\left(E_8^B\right) = \underbrace{\alpha^{-1}\xi_9 E_8^{LN}}_{\text{LN Efflux}} - \underbrace{\mu_3 E_8^B}_{\text{Natural death}} - \sum_{g \in Granulomas} Pulled\left[E_8^B\right]_g$$

CM_8^B - BlCM8 - Mtb-specific central memory CD8$^+$ T-cell count

$$\frac{d}{dt}\left(CM_8^B\right) = \underbrace{\alpha^{-1}\xi_{11} CM_8^{LN}}_{\text{LN Efflux}} - \underbrace{\xi_{10} CM_8^B}_{\text{LN Influx}} - \underbrace{k_{17} CM_8^B \left(\frac{APC}{APC + hs_{17}}\right)}_{\text{Cytokine-mediated recruitment}}$$

EM_8^B - BlEM8 - Mtb-specific Effector memory CD8$^+$ T-cell count

$$\frac{d}{dt}\left(EM_8^B\right) = \underbrace{\alpha^{-1}\xi_{12} EM_8^{LN}}_{\text{LN Efflux}} - \underbrace{\mu_4 EM_8^B}_{\text{Natural death}} - \sum_{g \in Granulomas} Pulled\left[EM_8^B\right]_g$$

$N_{Non,8}^B$ - BlN8Non - Non-cognate naive CD8$^+$ T-cell count

$$\frac{d}{dt}\left(N_{Non,8}^B\right) = \underbrace{(1-\lambda)\, s_{N_8}}_{\text{Source from thymus}} + \underbrace{\alpha^{-1}\xi_8 N_{Non,8}^{LN}}_{\text{LN Efflux}}$$
$$- \underbrace{k_{10} N_{Non,8}^B \left(\frac{APC}{APC + hs_{10}}\right)}_{\text{Cytokine-mediated recruitment}} - \underbrace{\xi_7 N_{Non,8}^B}_{\text{LN Influx}} - \underbrace{\mu_9 N_{Non,8}^B}_{\text{Natural death}}$$

$E_{Non,8}^B$ - BlE8Non - Non-cognate effector CD8$^+$ T-cell count

$$\frac{d}{dt}\left(E_{Non,8}^B\right) = \underbrace{s_{E_{Non,8}}}_{\text{Source from thymus}} - \underbrace{\mu_3 E_{Non,8}^B}_{\text{Natural death}} \sum_{g \in Granulomas} Pulled\left[E_{Non,8}^B\right]_g$$

$CM_{Non,8}^B$ - BlCM8Non - Non-cognate central memory CD8$^+$ T-cell count

$$\frac{d}{dt}\left(CM_{Non,8}^B\right) = \underbrace{\alpha^{-1}\xi_{11} CM_{Non,8}^{LN}}_{\text{LN Efflux}} - \underbrace{\xi_{10} CM_{Non,8}^B}_{\text{LN Influx}} - \underbrace{k_{17} CM_{Non,8}^B \left(\frac{APC}{APC + hs_{17}}\right)}_{\text{Cytokine-mediated recruitment}}$$

$EM_{Non,8}^B$ - BlEM8Non - Non-cognate effector memory CD8$^+$ T-cell count

$$\frac{d}{dt}\left(EM_{Non,8}^B\right) = \underbrace{s_{EM_{Non,8}}}_{\text{Source from thymus}} - \underbrace{\mu_4 EM_{Non,8}^B}_{\text{Natural death}} - \sum_{g \in Granulomas} Pulled\left[EM_{Non,8}^B\right]_g$$

3. Coupling granuloma equations to lymph node and blood equations

3.1. **Migration of APCs from granulomas to lymph nodes.** Each granuloma begins to send a portion of its infected macrophages to the lymph node compartment. We use infected macrophages M_I as a proxy for the number of dendritic cells that take Mtb to the lymph node. Each granuloma sends APCs to the lymph nodes after time τ_s under the assumption that granulomas do not immediately begin to send dendritic cells to the lymphatic system. We compute APCs sent by granuloma g to the lymph node as:

$$APC \text{ Sent by Granuloma } g \text{ at time } t \text{ post-founding} = \begin{cases} 0 & \text{if } t < \tau_s \\ A \cdot M_I & \text{otherwise.} \end{cases}$$

3.2. **Granulomas requests T-cells, and pulls from the blood compartment.** In *HostSim*, a given host begins with 13 granulomas. As infection progresses, each granuloma will begin to attempt to recruit six types of T-cells from the blood compartment: $T_0^4, T_0^8, T_{EM}^4, T_{EM}^8, T_{Non}^4$, and T_{Non}^8. At each time step, each granuloma will independently request a number of T-cells given below:

- Requests for T_0^4 :

$$Requested\left[T_0^4\right] = \underbrace{\alpha_{1a}\left(w_2 M_I + M_A\right)}_{\text{Macrophage-mediated request}} + \underbrace{Sr_{1b}\left(\frac{F_\alpha}{F_\alpha + f_8 I_{10} + s_{4b2}}\right)}_{\text{TNF-mediated request}}$$

- Requests for T_0^8 :

$$Requested\left[T_0^8\right] = \underbrace{\alpha_{1a}\left(M_A + w_2 M_I\right)}_{\text{Macrophage-mediated recruitment}} + \underbrace{Sr_{1b}\left(\frac{F_\alpha}{F_\alpha + f_8 I_{10} + s_{4b2}}\right)}_{\text{TNF mediated recruitment}}$$

- Requests for T_{EM}^4:

$$Requested\left[T_{EM}^4\right] = \underbrace{Sr_{4EM}\left(\frac{F_\alpha}{F_\alpha + c_{4EM}}\right)}_{\text{TNF driven recruitment}}$$

- Requests for T_{EM}^8:

$$Requested\left[T_{EM}^8\right] = \underbrace{Sr_{8EM}\left(\frac{F_\alpha}{F_\alpha + hs_{8EM}}\right)}_{\text{TNF-mediated recruitment}}$$

- Requests for T_{Non}^4:

$$Requested\left[T_{Non}^4\right] = \underbrace{Sr_{4Non}\left(\frac{F_\alpha}{F_\alpha + hs_{4Non}}\right)}_{\text{TNF driven recruitment}}$$

- Requests for T_{Non}^8:

$$Requested\left[T_{Non}^8\right] = \underbrace{Sr_{8Non}\left(\frac{F_\alpha}{F_\alpha + hs_{8Non}}\right)}_{\text{TNF driven recruitment}}$$

The blood's response to these requests is to provide lung each granuloma with T-cells. Since we model the T-cell subpopulations in more granularity in the lymph node and blood compartments, we associate the following populations between the terminology of the lymph node and blood equations; and the granuloma equations.

(1) Granuloma requests for T_0^4 pull from Lymph/blood concentration E_4^B.
(2) Granuloma requests for T_0^8 pull from Lymph/blood concentration E_8^B.
(3) Granuloma requests for T_{EM}^4 pull from Lymph/blood concentration EM_4^B.
(4) Granuloma requests for T_{EM}^8 pull from Lymph/blood concentration EM_8^B.
(5) Granuloma requests for T_{Non}^4 pull from both Lymph/blood concentrations $E_{Non,4}^B$ and $EM_{Non,4}^B$.
(6) Granuloma requests for T_{Non}^8 pull from both Lymph/blood concentrations $E_{Non,8}^B$ and $EM_{Non,8}^B$.

For T_0^4, T_0^8, T_{EM}^4, and T_{EM}^8, the amount that is pulled from the blood to provide to granuloma g is given by:

$$\underbrace{Pulled\left[T^*\right]_g}_{T\text{ - Cell count}} = \alpha \cdot \underbrace{Pulled\left[E^*\right]_g}_{E\text{ - Concentration}}$$

$$= \begin{cases} Requested\left[T^*\right]_g & \text{if } \sum_{\text{Granulomas } j} Requested\left[T^*\right]_j < \alpha \cdot E^* \\ Relative\ Request\left[T^*\right]_g & \text{otherwise.} \end{cases}$$

Here, if there are not enough T-cells in the blood to meet the amount requested by the lung granulomas, available T-cells will be divided among the requesting granulomas by using the following relative request. For granuloma g, its relative request is

$$\mathit{Relative\ Request}\left[T^*\right]_g = \frac{\alpha \cdot E^*}{\sum_{\text{All granulomas}j} \mathit{Request}\left[T^*\right]_j}.$$

For T^4_{Non} and T^8_{Non}, we combine their two associated blood concentrations. For example,

$$\begin{aligned}\mathit{Pulled}\left[T^4_{Non}\right]_g &= \alpha\left(\mathit{Pulled}\left[E^B_{Non,4}\right]_g + \mathit{Pulled}\left[EM^B_{Non,4}\right]_g\right)\\ &= \begin{cases}\mathit{Requested}\left[T^4_{Non}\right]_g & \text{if } \sum_{\text{Granulomas } j} \mathit{Requested}\left[T^4_{Non}\right]_j < \alpha\left(E^B_{Non,4} + EM^B_{Non,4}\right)\\ \mathit{Relative\ Request}\left[T^4_{Non}\right]_g & \text{otherwise}\end{cases}.\end{aligned}$$

If the blood has excess T-cells ($E^B_{Non,4}$ and $EM^B_{Non,4}$ in this case), the weight of their contribution to the granulomas is based on their current relative population:

$$\alpha \cdot \mathit{Pulled}\left[E^B_{Non,4}\right]_g = \mathit{Requested}\left[T^4_{Non}\right]_g \frac{E^B_{Non,4}}{\left(E^B_{Non,4} + EM^B_{Non,4}\right)};$$

$$\alpha \cdot \mathit{Pulled}\left[EM^B_{Non,4}\right]_g = \mathit{Requested}\left[T^4_{Non}\right]_g \frac{EM^B_{Non,4}}{\left(E^B_{Non,4} + EM^B_{Non,4}\right)}.$$

If there are not enough T-cells for the request, the relative requests are calculated similarly and the blood concentration is exhausted:

$$\alpha \cdot \mathit{Pulled}\left[E^B_{Non,4}\right]_g = \frac{\mathit{Requested}\left[T^4_{Non}\right]_g}{\sum_{\text{Granulomas } j} \mathit{Requested}\left[T^4_{Non}\right]_j} \frac{E^B_{Non,4}}{\left(E^B_{Non,4} + EM^B_{Non,4}\right)};$$

$$\alpha \cdot \mathit{Pulled}\left[EM^B_{Non,4}\right]_g = \frac{\mathit{Requested}\left[T^4_{Non}\right]_g}{\sum_{\text{Granulomas } j} \mathit{Requested}\left[T^4_{Non}\right]_j} \frac{EM^B_{Non,4}}{\left(E^B_{Non,4} + EM^B_{Non,4}\right)}.$$

Pulled concentrations for T^8_{Non} are calculated in the same way.

4. Dissemination

In *HostSim*, nonsterile granulomas have a chance of disseminating. Each granuloma begins with a local dissemination probability adjustment L_t and nonlocal dissemination probability adjustment N_t when the granuloma is created (either primary at $t = 0$, or at some later time). At each timestep, a dissemination event is determined randomly determining:

$$\{\text{Local dissemination occurs at time } t\} = (L_t + U[0,1]) > 1$$

and

$$\{\text{Nonlocal dissemination occurs at time } t\} = (N_t + U[0,1]) > 1$$

At each time step, the probability adjustment of local dissemination increases:

$$L_t = L_{t-1} + \lambda_{local}\left(\frac{\left[B_I + B_E\right](t)}{\left[B_I + B_E\right](t) + k_{local}}\right).$$

Similarly, nonlocal dissemination probability adjustment builds as

$$N_t = N_{t-1} + \lambda_{nonlocal}\left(\frac{\left[B_I + B_E\right](t)}{\left[B_I + B_E\right](t) + k_{nonlocal}}\right).$$

A new locally-disseminated granuloma is created with its parameters selected to be normally distributed around the parameter values of the primary granuloma that it disseminated from (with $\sigma = 10\%$ of parent value). This is because of the assumption that nearby lung tissue conditions give rise to similar dynamics.

Nonlocally disseminated granlomas are given parameters uniformly sampled out of the ranges presented in Section 5.

Disseminated granulomas begin with one infected macrophage and an average number of intracellular bacteria from its parent (B_I/M_I from the primary granuloma that seeded it).

5. Parameter Ranges

5.1. **Granuloma parameter ranges.** Note that the rate constants k_i in the granuloma compartment and LN/blood compartment are different.

Parameter	Non-symbolic	Units	Description	Minimum Value	Maximum Value
s_{rm}	Srm	1/day	M_R recruitment rate	0	0
α_{4a}	alpha4a	1/day	Macrophage-driven M_R recruitment rate	0.7246	0.9097
β	Beta	1/pg	Scaling factor for F_α for M_R activation	8652476.9683	11390938.3080
w	w	-	Contribution of B_I to M_R recruitment	0.2601	0.3632
w_2	w2	-	Contribution of M_I to M_R recruitment	0.9572	1.1725
w_3	w3	-	Contribution of T_1^4 to M_I apoptosis	0.1	0.5
Sr_{4b}	Sr4b	1/day	F_α recruitment rate of M_R	61.6952	947.9850
f_8	f8	-	Ratio adjustment of I_{10}/F_α on M_R recruitment	0.02568	0.9748
f_9	f9	-	Ratio adjustment of I_{10}/F_α for apoptosis rates	0.1	1.0
s_{4b}	s4b	pg/mL	Half saturation of F_α on M_R recruitment	48.02390	427.4012
k_4	k4	1/day	M_A decativation by I_{10}	0.08308	0.1599
s_8	s8	pg/mL	Half saturation of I_{10} on M_A deactivation	118.2688	536.2867
k_2	k2	1/day	M_R infection rate	0.1182	0.5718
c_9	c9	count	Half saturation of B_E on M_R infection	1468.08355	9039.5524
k_3	k3	1/day	M_R activation rate	0.1134	0.4659
f_1	f1	-	Ratio adjustment of I_4/I_γ on M_R activation	132.009929	477.7016
s_1	s1	pg/mL	Half saturation of I_γ dependent M_R activation	2.6104	97.4874
c_8	c8	count	Half saturation of B_E and B_I on M_R activation	3455.3760	6811.6706
μ_{M_R}	muMR	1/day	M_R death rate	0.004432	0.005695
k_{17}	k17	1/day	Maximum rate of M_I bursting	0.1048	0.2273
N	N	count	Carrying capacity of Mtb in one M_I	6.8186	26.3527
k_{14a}	k14a	1/day	T-cell induced apoptosis rate of M_I	0.3029	0.5
c_4	c4	count	Half saturation of T_1^4/M_I on T-cell apoptosis	1805.8568	9420.9084
k_{14b}	k14b	1/day	F_α-induced apoptosis rate	0.2478	0.4838
k_{52}	k52	1/day	Cytotoxic killing rate of M_I	0.09547	0.3910
w_1	w1	-	Contribution of T_1^4 to cytotoxic killing	0.2192	0.7413
c_{62}	c52	count	Half-saturation of T_C on cytotoxic M_I killing	2588.9284	8088.5625
$C_{T_1^4}$	cT1	count	Half saturation of T_1^4 on cytotoxic killing	22.6446	88.4901
μ_{M_I}	muMI	1/day	M_I death rate	0.002965	0.003799
μ_{M_A}	muMA	1/day	M_A death rate	0.05	0.1
α_{1a}	alpha1a	1/day	Macrophage request rate of T_0^4	0.1769	0.4336

Parameter	Non-symbolic	Units	Description	Minimum Value	Maximum Value
Sr_{1b}	Sr1b	1/day	F_α dependent T_0^4 recruitment	25767.7947	53329.7803
s_{4b2}	s4b2	pg/mL	Half saturation of F_α dependent T_0^4 recruitment	996.03150	8822.9850
α_2	alpha2	1/day	Maximum growht rate of T_0^4	0.04446	0.9666
c_{15}	c15	count	Half saturation of M_A proliferation of T_0^4	531.8574	9574.4150
k_6	k6	1/day	Maximum T_0^4 to T_1^4 differentiation rate	0.003726	0.09727
f_7	f7	-	Effect of I_{10} on I_γ induced $T_0^4 \rightarrow T_1^4$ differentiation	6.2748	44.6670
k_7	k7	1/day	Maximum rate of $T_0^4 \rightarrow T_2^4$ differentiation	0.2437	0.6125
f_2	f2	-	Ratio adjustment of I_γ/I_4 on $T_0^4 \rightarrow T_2^4$ differentiation	0.1938	0.4207
s_2	s2	pg/mL	Half saturation of I_4 for $T_0^4 \rightarrow T_2^4$ differentiation	36.3818	964.1090
μ_{T_0}	muT0	1/day	$CD3^+$ primed T-cell death rate	0.1957	0.2474
m	m	-	Fraction of differentiating T_0^8 that become T^8 (and not T_C)	0.1364	0.8643
μ_{T_g}	muTg	1/day	T_C death rate	0.005308	0.09570
c	c	pg/mL	Half saturation of I_γ on T_1^4 apoptosis	462.9043	9539.3955
$\mu_{T_1^4}$	muT1	1/day	T_1^4 death rate	0.2848	0.3698
$\mu_{T_2^4}$	muT2	1/day	T_2^4 death rate	0.2913	0.3678
μ_{TC_γ}	muTCg	1/day	I_γ driven apoptosis rate of T_C and T^8	0.006021	0.09506
c_c	cc	pg/mL	Half saturation of I_γ on T_C and T^8 apoptosis	368.2888	9634.1767
μ_{T_C}	muTC	1/day	T_C death rate	0.2570	0.3325
α_{30}	alpha30	1/day	Rate of F_α production by M_I	0.04474	0.09741
α_{31}	alpha31	1/day	Rate of F_α production by M_A	0.05071	0.09656
β_2	beta2	1/pg	Scaling factor of Mtb on F_α production by M_A	10944.8638	13022.3032
s_{10}	s10	pg/mL	Half saturation of I_γ on F_α production by M_A	102.8084	313.4010
α_{32}	alpha32	pg/(mL*day)	F_α production rate by T_1^4	0.2048	0.3447
α_{33}	alpha33	pg/(mL*day)	F_α production rate by T^8 and T_C	0.1815	0.3330
μ_{TNF}	muTNF	1/day	F_α decay rate	0.9343	1.2101
s_g	sg	pg/(mL*day)	I_γ production from other sources (e.g. dendritic cells)	846.3332	5484.1532
c_{10}	c10	count	Half saturation of Mtb production from other sources	315332.8289	536623.8464
s_7	s7	pg/mL	Half saturation of I_{12} on I_γ production from other sources	591.2493	851.7672
α_{5a}	alpha5a	pg/day	Rate of I_γ production by T_1^4	0.5443	0.8698
c_{5a}	c5a	count	Half saturation of M_A on I_γ production by T_1^4	303.7365	687.08808
α_{5b}	alpha5b	pg/day	I_γ production by T^8	1.1445	15.1796
α_{5c}	alpha5c	pg/day	I_γ production by M_I	0.5192	0.9184
c_{5b}	c5b	count	Half saturation of M_A on I_γ production by T^8	235.8238	846.5511
α_7	alpha7	pg/day	I_γ production by T_0^4	0.08541	0.3098
f_4	f4	-	Adjustment of I_{10}/I_{12} on I_γ production	1.2966	1.6715
s_4	s4	pg/mL	Half saturation of I12 on I_γ	321.7448	865.2110
μ_{I_γ}	muIG	1/day	I_γ decay rate	5.4475	9.6934
α_{23}	alpha23	pg/day	Rate of I_{12} production by M_R	0.003485	0.004652
c_{23}	c23	count	Half saturation of Mtb on I_{12} production by M_R	157.08445	525.4198

Parameter	Non-symbolic	Units	Description	Minimum Value	Maximum Value
α_8	alpha8	pg/day	Rate of production of I_{12} by M_A	0.3764	0.8612
s_{12}	s12	pg/day	Rate of production of I_{12} by dendritic cells	2361.1941	4060.7654
c_{230}	c230	count	Half-saturation of Mtb in I_{12} production by DCs	365.6847	761.7284
$\mu_{I_{12}}$	muI12	1/day	I_{12} decay rate	0.9319	1.2420
s	s	pg/mL	I_{10} effect on I_{12} production by M_A	191.7181	694.3849
δ_7	delta7	pg/day	I_{10} production by M_A	0.1106	0.6169
s_6	s6	pg/mL	Half saturation of I_{10} on self	587.3834	858.9373
f_6	f6	-	Ratio adjustment of I_γ on I_{10}	0.3011	0.3889
α_{16}	alpha16	pg/day	I_{10} production by T_1^4	0.4280	0.6693
α_{17}	alpha17	pg/day	I_{10} production by T_2^4	0.4149	0.4786
α_{18}	alpha18	pg/day	I_{10} production by T_C and T^8	0.5184	0.6648
$\mu_{I_{10}}$	muI10	1/day	I_{10} decay rate	0.5064	4.1388
α_{11}	alpha11	pg/day	I_4 production by T_0^4	0.02944	0.06404
α_{12}	alpha12	pg/day	I_4 production by T_2^4	0.02110	0.06407
μ_{I_4}	muI4	1/day	I_4 decay rate	2.3700	3.08806
α_{19}	alpha19	1/day	B_I growth rate	0.8739	1.3173
α_{20}	alpha20	1/day	B_E growth rate	0.4190	0.5828
N_{fracc}	Nfracc	-	Fraction of surviving B_I released by T-cell M_I apop.	0.3074	0.7893
N_{fraca}	Nfraca	-	Fraction of surviving B_I released by TNF M_I apop.	0.2680	0.5658
k_{15}	k15	1/day	B_E killing rate by M_A	0.03891	0.09025
k_{18}	k18	1/day	B_E killing rate by M_R	0.0003264	0.0005505
N_{fracd}	Nfracd	-	Fraction of surviving B_I released by M_I natural death.	0.0008721	0.001098
μ_{B_I}	muBI	1/day	B_I death rate	0.00005947	0.00009042
μ_{B_E}	muBE	1/day	B_E death rate	0.000000004107	0.000000007405
Sr_{4Non}	Sr4Non	1/day	Rate of TNF-driven T_{Non}^4 recruitment	155.6978	504.2867
hs_{4Non}	hs4Non	pg/mL	Half saturation of TNF in T_{Non}^4 recruitment	5.6995	50.1242
μ_{4Non}	mui4Non	1/day	Death rate of T_{Non}^4	0.2631	0.3579
Sr_{8Non}	Sr8Non	1/day	Rate of TNF-driven T_{Non}^8 recruitment	153.04868	508.8051
hs_{8Non}	hs8Non	pg/day	Half saturation of TNF in T_{Non}^8 recruitment	4.09874	40.7487
$\mu_{T_{Non}^8}$	mui8Non	1/day	Death rate of T_{Non}^8	0.2644	0.3543

Parameter	Non-symbolic	Units	Description	Minimum Value	Maximum Value
Sr_{4EM}	Sr4EM	1/day	Rate of TNF-driven T^4_{EM} recruitment	49.1138	100.03829
hs_{4EM}	hs4EM	pg/mL	Half saturation of TNF in T^4_{EM} recruitment	0.001	0.001
$\mu_{T^4_{EM}}$	mui4EM	1/day	Death rate of T^4_{EM}	0.001368	0.002737
k_{31}	k31	1/day	Differentiation rate of $T^4_{EM} \to T^4_1$	0.005592	0.1
k_{32}	k32	1/day	Differentiation rate of $T^4_{EM} \to T^4_2$	0.0006898	0.001086
Sr_{EM^8}	Sr8EM	1/day	Rate of TNF-driven T^8_{EM} recruitment	54.7352	105.4204
hs_{EM^8}	hs8EM	pg/mL	Half saturation of TNF in T^8_{EM} recruitment	0.001	0.001
$\mu_{T^8_{EM}}$	mui8EM	1/day	Death rate of T^8_{EM}	0.001368	0.002737
k_{33}	k33	1/day	Differentiation rate of $T^8_{EM} \to T_C$	0.006339	0.1
$k34$	k34	1/day	Differentiation rate of $T^8_{EM} \to T^8$	0.005329	0.1
τ_s	taus	days	Time that granuloma begins sending APCs to LN	4.02618	22.1392
A	APCpercent	-	% of M_I that get sent as APCs	0.1723	0.2893
k_{local}	localDissemCFUHalf	count	Half-saturation of Mtb for local dissemination	10000.0	50000.0
λ_{local}	localDissemLambda	1/day	Maximum rate of local dissemination	0.005	0.025
$k_{nonlocal}$	nonLocalDissemCFUHalf	count	Half-saturation of Mtb for nonlocal dissemination	5223.04303	10608.01033
$\lambda_{nonlocal}$	nonLocalDissemLambda	1/day	Maximum rate of nonlocal dissemination	0.001	0.01
μ_{B_N}	muBN	1/day	Death rate of B_N	0.04032	0.06819
C_N	CN	-	Fraction of released B_I that become B_N	0.1254	0.1725
N_f	Nf	Mϕ/caseum	Macrophage biomass that becomes caseum	2.09563	3.9056
N_{Ca}	Nca	1/(day*caseum)	Clearance rate of caseum by neutrophils	0.000001273	0.000008516

Initial Condition	Non-Symbolic	Units	Description	Min initial value	Max inial value
$M_R(0)$	MR	count	Resting macrophages	1.0	5.0
$M_I(0)$	MI	count	Infected macrophages	1.0	1.0
$M_A(0)$	MA	count	Activated macrophages	1.0	1.0
$B_I(0)$	BI	count	Intracellular Mtb	1.0	1.0
$B_E(0)$	BE	count	Extracellular Mtb	0	0
$B_N(0)$	BN	count	Non-replicating Mtb	0	0
$T_0^4(0)$	T40	count	Mtb-specific Primed $CD4^+$ T-cells	0	0
$T_1^4(0)$	Th1	count	Mtb-specific Th1 $CD4^+$ T-cells	0	0
$T_{Non}^4(0)$	CD4Non	count	Nonspecific $CD4^+$ T-cells	0	0
$T_2^4(0)$	Th2	count	Mtb-specific Th2 $CD4^+$ T-cells	0	0
$T_{EM}^8(0)$	EMCD4	count	Mtb-specific $CD4^+$ effector memory T-cells	0	0
$T_0^8(0)$	T80	count	Mtb-specific Primed $CD8^+$ T-cells	0	0
$T_C(0)$	TC	count	Mtb-specific Cytotoxic $CD8^+$ T-cells	0	0
$T^8(0)$	T8	count	Mtb-specific Effector $CD8^+$ T-cells	0	0
$T_{EM}^8(0)$	EMCD8	count	Mtb-specific $CD8^+$ effector memory T-cells	0	0
$T_{Non}^8(0)$	CD8Non	count	Nonspecific $CD8^+$ T-cells	0	0
$F_\alpha(0)$	TNF	pg/mL	TNF-α concentration	0	0
$I_\gamma(0)$	IG	pg/mL	IFN-γ concentration	0	0
$I_{12}(0)$	I12	pg/mL	Interleukin 12 concentration	0	0
$I_{10}(0)$	I10	pg/mL	Interleukin 10 concentration	0	0
$I_4(0)$	I4	pg/mL	Interleukin 4 concentration	0	0
$Ca(0)$	CA	mass of $M\phi$	Necrotic tissue mass	0	0

5.2. **Lymph Node and Blood parameter ranges.** Note that the rate constants k_i in the granuloma compartment and LN/blood compartment are different.

Parameter	non-symbolic	Units	Description	Lower range	Upper range
α	alpha	μL	Conversion from Blood concentration to LN cell counts	360000.0	360000.0
Host LNs	hostLn	count	Number of involved LNs in host (Used for initial conditions)	5.0	5.0
λ	lambda	-	Frequency of Mtb-specific naive cells in the host	0.0001	0.0001
hs_1	hs1	count	Half saturation of APC in N_4^B recruitment to LN	13.9295	70.7524
hs_{10}	hs10	count	Half saturation of APC in N_8^B recruitment to LN	45.5413	87.7386
hs_{11}	hs11	count	Half saturation of CD4$^+$ surrogates in N_8^{LN} priming	12.9592	47.9200
hs_{13}	hs13	count	Half saturation of APC in P_8^{LN} proliferation	2684.2696	4055.8241
hs_{14}	hs14	count	HS of APC in $P_8^{LN} \to E_8^{LN}$ and $P_8^{LN} \to CM_8^{LN}$differentiation	1904.3871	4144.4838
hs_{17}	hs17	count	Half saturation of APC in CM_8^B recruitment to LN	66.1225	403.04107
hs_4	hs4	count	Half saturation of APC in P_4^{LN} proliferation	1318.9532	4317.6863
hs_5	hs5	count	HS of APC in $P_4^{LN} \to E_4^{LN}$ and $P_4^{LN} \to CM_4^{LN}$ differentiation	1257.1402	3718.5646
hs_8	hs8	count	Half saturation of APC in $CM_{Non,4}^B$ recruitment to LN	40.4710	57.04244
k_1	k1	1/day	Rate of N_4^B recruitment to LN	0.1156	0.4736
k_{10}	k10	1/day	Rate of N_8^B recruitment to LN	0.7686	0.9663
k_{11}	k11	1/day	Rate of N_8^{LN} priming	0.0001043	0.0002272
k_{12}	k12	1/day	CM_8^{LN} reactivation rate	0.0001206	0.0007482
k_{13}	k13	1/day	Rate of P_8^{LN} proliferation	0.1961	0.7954
k_{14}	k14	1/day	Rate of $P_8^{LN} \to E_8^{LN}$ differentiation	0.2548	0.7351
k_{15}	k15	1/day	Rate of $P_8^{LN} \to CM_8^{LN}$ differentiation	0.5286	0.8602
k_{16}	k16	1/day	Rate of $E_8^{LN} \to EM_8^{LN}$ differentiation	0.1573	0.8217
k_{17}	k17	1/day	Rate of CM_8^B recruitment to LN	0.3483	0.9012
k_2	k2	1/day	Rate of $N_4^{LN} \to P_4^{LN}$ differentiation	0.3279	0.8656
k_3	k3	1/day	Rate of $CM_4^{LN} \to P_4^{LN}$ differentiation	0.02161	0.07979
k_4	k4	1/day	Rate of P_4^{LN} proliferation	0.3131	1.3564
k_5	k5	1/day	Rate of $P_4^{LN} \to E_4^{LN}$ differentiation	0.2721	0.8993
k_6	k6	1/day	Rate of $P_4^{LN} \to CM_4^{LN}$ differentiation	0.3110	0.8645
k_7	k7	1/day	Rate of $E_4^{LN} \to EM_4^{LN}$ differentiation	0.1493	0.5799
k_8	k8	1/day	Rate of CM_4^B recruitment to LN	0.02620	0.06984

Parameter	Non-symbolic	Units	Description	Lower range	Upper range
μ_1	mu1	1/day	Death rate of E_4^B	0.2	0.2
μ_2	mu2	1/day	Death rate of EM_4^B	0.001368	0.002737
μ_3	mu3	1/day	Death rate of E_8^B	0.2	0.2
μ_4	mu4	1/day	Death rate of EM_8^B	0.001368	0.002737
μ_5	mu5	1/day	Death rate of APC	0.05	0.05
μ_6	mu6	1/day	Death rate of P_4^{LN}	0.0005	0.0005
μ_7	mu7	1/day	Death rate of P_8^{LN}	0.015	0.015
μ_8	mu8	1/day	Death rate of N_4^B	0.3	0.3
μ_9	mu9	1/day	Death rate of μ_9^{LN}	0.05	0.05
ρ_1	rho1	count	Precursor cell carrying capacity	300000000.0	300000000.0
w_{P_4}	Wp4	-	Contribution of P_4^{LN} in P_8^{LN} priming	0.7355	0.7355
ξ_{11}	xi11	1/day	$CM_8^{LN} \to CM_8^B$ LN efflux rate	0.2750	1.2233
ξ_{12}	xi12	1/day	$EM_8^{LN} \to EM_8^B$ LN efflux rate	0.2190	1.5214
ξ_2	xi2	1/day	$N_4^{LN} \to N_4^B$ LN efflux rate	1.9942	4.5066
ξ_3	xi3	1/day	$E_4^{LN} \to E_4^B$ LN efflux rate	0.9937	3.6017
ξ_5	xi5	1/day	$CM_4^{LN} \to CM_4^B$ LN Efflux rate	1.07179	3.9185
ξ_6	xi6	1/day	$EM_4^{LN} \to EM_4^B$ LN Efflux rate	2.5675	3.9231
ξ_8	xi8	1/day	$N_8^{LN} \to N_8^B$ LN Efflux rate	0.6344	2.2140
ξ_9	xi9	1/day	$E_8^{LN} \to E_8^B$ LN Efflux rate	1.8734	4.4935
ξ_1	xi1	1/day	$N_4^B \to N_4^{LN}$ LN Influx rate	0.3988	0.9013
ξ_4	xi4	1/day	$CM_4^B \to CM_4^{LN}$ LN Influx rate	0.2143	0.7837
ξ_7	xi7	1/day	$N_8^B \to N_8^{LN}$ LN Influx rate	0.1268	0.4428
ξ_{10}	xi10	1/day	$CM_8^B \to CM_8^{LN}$ LN Influx rate	0.05500	0.2446
s_{N_4}	Sn4	1/day*μL	Production rate of naive CD4$^+$ T-cells	20.1207	47.9125
s_{N_8}	Sn8	1/day*μL	Production rate of naive CD8$^+$ T-cells	0.3830	0.4868
$s_{E_{Non,4}}$	Senc4	1/day*μL	Production rate of effector non-specific CD4$^+$ T-cells	15.3195	65.7082
$s_{EM_{Non,4}}$	Semnc4	1/day*μL	Production rate of effector memory non-specific CD4$^+$ T-cells	6.4041	17.04971
$s_{E_{Non,8}}$	Senc8	1/day*μL	Production rate of effector non-specific CD8$^+$ T-cells	10.05032	69.3024
$s_{EM_{Non,8}}$	Semnc8	1/day*μL	Production rate of effector memory non-specific CD8$^+$ T-cells	2.8856	5.5002

Initial condition	Non-Symbolic	Unit	Description	Lower range	Upper range
$APC(0)$	APC	count	Antigen-presenting cells in LN	0	0
$N_4^{LN}(0)$	LnN4	count	Mtb-specific naive CD4$^+$ T-cell count in LN	2897.3857	6899.4095
$P_4^{LN}(0)$	LnP4	count	Mtb-specific precursor CD4$^+$ T-cell count in LN	0	0
$E_4^{LN}(0)$	LnE4	count	Mtb-specific effector CD4$^+$ T-cell count in LN	0	0
$CM_4^{LN}(0)$	LnCM4	count	Mtb-specific central memory CD4$^+$ T-cell count in LN	0	0
$EM_4^{LN}(0)$	LnEM4	count	Mtb-specific effector memory CD4$^+$ T-cell count in LN	0	0
$N_4^{B}(0)$	BlN4	pg/mL	Mtb-specific naive CD4$^+$ T-cell count in blood	*	-
$E_4^{B}(0)$	BlE4	pg/mL	Mtb-specific effector CD4$^+$ T-cell count in blood	*	-
$CM_4^{B}(0)$	BlCM4	pg/mL	Mtb-specific central memory CD4$^+$ T-cell count in blood	*	-
$EM_4^{B}(0)$	BlEM4	pg/mL	Mtb-specific effector memory CD4$^+$ T-cell count in blood	*	-
$N_8^{LN}(0)$	LnN8	count	Mtb-specific naive CD8$^+$ T-cell count in LN	5515.3085	7010.3557
$P_8^{LN}(0)$	LnP8	count	Mtb-specific precursor CD8$^+$ T-cell count in LN	0	0
$E_8^{LN}(0)$	LnE8	count	Mtb-specific effector CD8$^+$ T-cell count in LN	0	0
$CM_8^{LN}(0)$	LnCM8	count	Mtb-specific central memory CD8$^+$ T-cell count in LN	0	0
$EM_8^{LN}(0)$	LnEM8	count	Mtb-specific effector memory CD8$^+$ T-cell count in LN	0	0
$N_8^{B}(0)$	BlN8	pg/mL	Mtb-specific naive CD8$^+$ T-cell count in blood	*	-
$E_8^{B}(0)$	BlE8	pg/mL	Mtb-specific effector CD8$^+$ T-cell count in blood	*	-
$CM_8^{B}(0)$	BlCM8	pg/mL	Mtb-specific central memory CD8$^+$ T-cell count in blood	*	-
$EM_8^{B}(0)$	BlEM8	pg/mL	Mtb-specific effector memory CD8$^+$ T-cell count in blood	*	-
$N_{Non,4}^{LN}(0)$	LnN4Non	count	Nonspecific naive CD4$^+$ T-cell count in LN	28970960.05181	68987195.6821
$CM_{Non,4}^{LN}(0)$	LnCM4Non	count	Nonspecific central memory CD4$^+$ T-cell count in LN	13572957.9382	32571421.8047
$N_{Non,4}^{B}(0)$	BlN4Non	pg/mL	Nonspecific naive CD4$^+$ T-cell count in blood	402.3744	958.1554
$E_{Non,4}^{B}(0)$	BlE4Non	pg/mL	Nonspecific effector CD4$^+$ T-cell count in blood	76.5979	328.5413
$CM_{Non,4}^{B}(0)$	BlCM4Non	pg/mL	Nonspecific central memory CD4$^+$ T-cell count in blood	188.5133	452.3808
$EM_{Non,4}^{B}(0)$	BlEM4Non	pg/mL	Nonspecific effector memory CD4$^+$ T-cell count in blood	160.1049	426.2429
$N_{Non,8}^{LN}(0)$	LnN8Non	count	Nonspecific naive CD8$^+$ T-cell count in LN	55147570.5476	70096547.5890
$CM_{Non,8}^{LN}(0)$	LnCM8Non	count	Nonspecific central memory CD8$^+$ T-cell count in LN	8169880.9373	30928893.4342
$N_{Non,8}^{B}(0)$	BlN8Non	pg/mL	Nonspecific naive CD8$^+$ T-cell count in blood	765.9384	973.5631
$E_{Non,8}^{B}(0)$	BlE8Non	pg/mL	Nonspecific effector CD8$^+$ T-cell count in blood	50.2516	346.5122
$CM_{Non,8}^{B}(0)$	BlCM8Non	pg/mL	Nonspecific central memory CD8$^+$ T-cell count in blood	113.4705	429.5679
$EM_{Non,8}^{B}(0)$	BlEM8Non	pg/mL	Nonspecific effector memory CD8$^+$ T-cell count in blood	160.3165	305.5719

*We assume that at $t = 0$, blood and lymph node concentrations are at equilibrium, so blood concentrations are calculated via their lymph-node starting count via the conversion factor α and HostLNs
