# Supplementary material for: A framework for multi-scale intervention modeling: virtual cohorts, virtual clinical trials, and model-to-model comparisons
Source: Front Syst Biol. 2024 Jan 22;3:1283341. doi: 10.3389/fsysb.2023.1283341 (PMC11415237; doi:10.3389/fsysb.2023.1283341)
Supplement: Supplementary file 4 [file DataSheet1.DOCX]

Supplementary Material 1

# Extended tuberculosis background

Infection occurs when *Mycobacterium tuberculosis* (Mtb) is inhaled and is not immediately cleared by the alveolar macrophages within lungs (the site of infection). Mtb prevents its digestion within macrophages and replicates slowly within phagosomes (>12 hour doubling time) until infected macrophages burst. These newly released Mtb are again phagocytosed by recruited macrophages, and this process repeats until at approximately 3-4 weeks post infection, when the adaptive immune system responds. Once effector T-cells arrive to the host's lungs and activate macrophages, they can efficiently kill Mtb. Granulomas, solid cellular immune structures that form in the lungs result from this infection process, have distinct regions: an inner, hypoxic, necrotic core comprising dead macrophages, neutrophils, and nonreplicating Mtb; a viable cellular zone where a battle is waged between Mtb (both intracellular within macrophages and neutrophils and extracellular) and a lymphocytic (T-cell) cuff. Granulomas do an excellent job containing infection, with most sterilizing or controlling bacteria to low levels (1, 2). Occasionally, Mtb will be carried from granulomas, seeding new granulomas, a process known as dissemination.

Each granuloma has its own infection progression trajectory, with more than half of granulomas sterilizing in a given host even if that host is experiencing active infection (1, 2). Even so, a single granuloma that cannot contain infection can result in severe infection (active infection) for the host and lead to death if not treated. Hosts that control the number of Mtb within granulomas at lower levels have clinically latent TB and can remain in this state (and potentially undiagnosed) for decades. However, if any of these granulomas later fail to contain the infection, e.g., due to immunosuppression, reactive TB is the result (3). We use total lung CFU counts at time of necropsy from four untreated NHPs with no noted comorbidities, from studies by JoAnne Flynn (4-7): 219740, 692813, 423300, and 57755 CFU, respectively. Each of these NHPs had to be necropsied before the study end time and are used to define the "active infection" threshold for classifying virtual patients, and so we use the median CFU value of 321520 to define our cutoff for active Mtb infection in virtual hosts. Our model is flexible to include any value for this threshold.

Host-scale dynamics (which hosts will clear, control, or suffer active disease) are heterogeneous within a population. This population scale heterogeneity may be influence by various factors. For example, a susceptibility mutation in HLA-DR2 makes populations more susceptible to disease (8). Additionally, lack of access to proper nutrition, clean water, or good sanitary practices may each increase population susceptibility. Finally, treatment protocols and vaccine campaigns at population scales can vary greatly by populations (1, 9, 10). To understand host infection progression and treatment, it is imperative to study TB at multiple scales, and elucidate how small-scale interactions influence large-scale findings (Figure 1).

# Single host simulation framework, *HostSim*, updates and calibration

## Model updates

# We expanded the functionality of *HostSim* in the present work in four ways: (i) inclusion of caseum within each of the multiple granulomas reflected in *HostSim*, (ii) inclusion of non-replicating bacteria within the caseum (Figure 2 B), and (iii) adding a carrying capacity for extracellular bacteria within each granuloma. Finally, (iv) we include the ability to study drug treatment in *HostSim*.

# When primates are infected with Mtb and granulomas form, dead cellular matter develops within the center of these structures due to cell death and killing. Bacteria can be trapped in this necrotic region, allowing them to survive for decades in a non-replicating state. The presence of caseum also makes treatment penetration within granulomas more difficult as drugs don't diffuse easily into this necrotic area (11, 12) We have newly included the formation of caseum within *HostSim* granulomas (Figure 2). Caseum is generated by the death of macrophages (which cell type also serve as a proxy for neutrophils, because we do not model them explicitly in *HostSim*). In *HostSim*, the number ($\boldsymbol{N}_{\boldsymbol{f}}$_﻿_﻿) of dead macrophages creates 1 unit of caseum. We allow this to vary between granulomas. The equation for caseum is given by:

$$N_{f}\frac{d}{dt}\left( Ca \right)=\underset{\text{Bursting of }M_{I}}{\underbrace{k_{17}M_{I}\left( \frac{B_{I}^{2}}{B_{I}^{2}+\left( NM_{I} \right)^{2}} \right)}}+\underset{\text{T-cell mediated apoptosis of }M_{I}}{\underbrace{k_{14a}M_{I}\left( \frac{\left( \frac{T_{C}+w_{3}T_{1}}{M_{I}} \right)}{\left( \frac{T_{C}+w_{3}T_{1}}{M_{I}} \right)+c_{4}} \right)}}+\underset{\text{TNF-mediated apoptosis of }M_{I}}{\underbrace{k_{14b}M_{I}\left( \frac{F_{\alpha}}{F_{\alpha}+f_{9}I_{10}+s_{4b}} \right)}}+\underset{\text{Cytotoxic T-cell mediated apoptosis of }M_{I}}{\underbrace{k_{52}\frac{B_{I}}{M_{I}}M_{I}\left( \frac{\left( \frac{T_{C}\left( \frac{T_{1}^{4}}{T_{1}^{4}+C_{T_{1}^{4}}} \right)+w_{1}T_{1}^{4}}{M_{I}} \right)}{\left( \frac{T_{C}\left( \frac{T_{1}^{4}}{T_{1}^{4}+C_{T_{1}^{4}}} \right)+w_{1}T_{1}^{4}}{M_{I}} \right)+c_{52}} \right)}}-\underset{\text{Neutrophil-driven caseum clearance}}{\underbrace{N_{Ca}CaM_{A}}}+\underset{\text{Natural death of all macrophage populations}}{\underbrace{\mu_{M_{I}}M_{I}+\mu_{M_{A}}M_{A}+\mu_{M_{R}}M_{R}\left( \frac{F_{\alpha}}{F_{\alpha}+f_{9}I_{10}+s_{4b}} \right)}}$$

﻿﻿﻿where the terms, in order, represent the distinct contribution of death of different immune cells to caseum: [1] bursting of infected macrophages, [2] T-cell induced apoptosis of infected macrophages, [3] TNF-induced apoptosis of infected macrophages, [4] cytotoxic T-cell induced apoptosis of infected macrophages, [5] cytotoxic T-cell mediated apoptosis of infected macrophages, and [6-8] natural death of infected, activated, and resting macrophages. Once this dead tissue appears within a granuloma it will not be cleared except very slowly by activated macrophages acting as a proxy for neutrophil behavior.

Now that we have included caseum within granulomas, we also allow for bacteria to become trapped within that region. Since Mtb are non-motile, once they are within the caseum compartment they will remain there. Dead tissue is hypoxic, and while the bacteria do not die within that region, they are also not able to grow and divide. We refer to these as non-replicating Mtb. Non-replicating Mtb are generated when Mtb are growing within infected macrophages and a macrophage is killed or bursts, it releases bacteria into the surrounding tissue. Since a macrophage has died, it will contribute to caseum, and a portion of the released bacterial load, $C_{N}$, will be trapped within caseum. By calibration, we note that calibration of $C_{N}$ to experimental accounts for the fact that not all macrophages are near the caseum. The non-replicating bacteria equation is:

$\frac{d}{dt}\left( B_{N} \right)=\underset{\text{Fraction inside of Caseum}}{\underbrace{C_{N}}}\left[ \underset{\text{Macrophage bursting}}{\underbrace{k_{17}M_{I}\left( \frac{B_{I}^{2}}{B_{I}^{2}+\left( NM_{I} \right)^{2}} \right)}}+\underset{\text{T-cell driven apoptosis}}{\underbrace{k_{14a}N_{fracc}\frac{B_{I}}{M_{I}}M_{I}\left( \frac{\left( \frac{T_{C}+w_{3}T_{1}^{4}}{M_{I}} \right)}{\left( \frac{T_{C}+w_{3}T_{1}^{4}}{M_{I}} \right)+c_{4}} \right)}}+\underset{\text{TNF driven apoptosis}}{\underbrace{k_{14b}N_{fraca}\frac{B_{I}}{M_{I}}M_{I}\left( \frac{F_{\alpha}}{F_{\alpha}+f_{9}I_{10}+s_{4b}} \right)}}+\underset{\text{Natural death of }M_{I}}{\underbrace{\mu_{M_{I}}N_{fracd}\frac{B_{I}}{M_{I}}M_{I}}} \right]-\underset{\text{Natural death}}{\underbrace{\mu_{B_{N}}B_{N}}}-\underset{\text{Revealing of }B_{N}}{\underbrace{B_{N}k_{Rev}N_{Ca}Ca\cdot M_{A}}}$﻿

where the terms, in order, represent non-replicating bacteria (1) fraction emerging from burst infected macrophages, (2-3) fraction emerging from infected macrophages dying via (2) T-cell or (3) TNF-induced apoptosis, (4) fraction emerging from naturally dying macrophages; (5) the natural death of non-replicating bacteria; and (6) non-replicating bacteria being exposed and becoming extracellular bacteria as a result of caseum clearance. The portion of the bacteria that survive to become trapped in caseum is affected by the mode of macrophage death. Parameters $N_{fraca}$_﻿_﻿, $N_{fracc}$_﻿﻿_﻿, and $N_{fracd}$﻿ capture this heterogeneity of bacteria survival based on mode of death (13).

To use *HostSim* to study drug treatment and vaccines, we updated our treatment of bacterial growth and our representation of extracellular Mtb _﻿_$B_{E}$_﻿_﻿. We updated Mtb growth from an exponential form to a logistic form, now given by the replication term $\alpha_{20}B_{E}\left( 1-B_{E}/{10}^{6} \right)$ replacing $\alpha_{20}B_{E}$_﻿_﻿. We included this to better capture the carrying capacity of individual granulomas (as we have done in *GranSim*, (14-17)), which have rarely been measured as containing more than ${10}^{6}$ CFUs (4-7). We also have included a source of extracellular Mtb, $B_{E}$ that are derived from the _﻿_$B_{N}$_﻿_﻿ that may be revealed by clean-up of caseum by cells such as neutrophils or once a macrophage bursts it may release the bacteria into extracellular spaces (18-21). A full listing of *HostSim* equations and parameters is given in Supplementary Material 2*.*

## Calibration criteria for granuloma and host scale parameters

*Experimental criteria: total Mtb CFU, macrophages, and T-cells per granuloma.* We make our assumptions based on NHP data. For example, each granuloma typically has no more than ${10}^{6}$_﻿_﻿ Mtb CFU. We assume this cap at any given time after day 90 of infection. Similarly, we use cell counts of macrophages and T-cells present within each granuloma at the time of necropsy from NHP data (4-7). Simulations in the ‘pass set’ were within one order of magnitude of total macrophage and total T-cell counts, shown in Supplementary Figure 1 A. In a similar way, we also ensured that the ratio of T-cells to macrophages in each granuloma lay strictly within the ratios observed from our experimental data set (4-7). Supplementary Figure 1 B shows resultant Mtb over time in *HostSim* compared to experimental data from NHPs (4-7).

*Synthetic criteria: caseum volume, macrophage ratios, and Mtb ratios.* We use our fine-grained hybrid agent-based model of single granuloma formation, *GranSim* (14-17), as a source of heuristic objectives in the absence of direct experimental data. In other words, we use our own synthetic data for calibrating caseum and other quantities not measurable from presently-available data. Since *GranSim* has been calibrated to a variety of temporal and spatial data over time with NHP data, we believe that using it to provide synthetic data is reasonable. The data we used includes several ratios of resting to activated macrophages, intracellular-to-extracellular Mtb, and the volume of the caseum within granulomas. Note that all measurable outcomes in *GranSim* (which is a two-dimensional agent-based model) are scaled to provide validated predictions of the three-dimensional granuloma (22).

To calibrate the amount of caseum in simulations, we first converted *GranSim* units of caseum to *HostSim* units of caseum by comparing the number of dead macrophages required to create caseum. This value is uniformly between 12 and 18 in *GranSim* (caseum in *GranSim* is described in (14)), and we represent this by using the average of 15 in the current work. In *HostSim*, this value is $1/N_{f}$_﻿_﻿, the caseation factor. We convert *GranSim* to *HostSim* caseum as:

$\left[ \text{GranSim}\text{ Caseum} \right] / \left[ \text{HostSim}\text{ }\text{Caseum} \right]=C_{N}/15\left( =N_{f}/15 \right)$._﻿﻿_

*HostSim* counts of non-replicating bacteria $B_{N}$ were low compared to those observed within *GranSim*. We believe that this is because a main source of $B_{N}$ in *GranSim* is from extracellular bacteria being trapped in forming caseum from nearby macrophages dying, which is more fine-grained and not represented in this iteration of *HostSim*.

In the LNs, previous calibration matched simulated and experimental levels of CD4^+^ and CD8^+^ total T-cell count, effector memory T-cell count, central memory cell count, and effector cell count (23). Having updated our LN calibration using our older parameter ranges as a starting point, our blood and lymph node T-cell populations still agree with blood data from the JoAnne Flynn lab (4-7), shown in Supplementary Figure 1 C-D.

# Model visualization

We plot the time-series for all variables in the model. However, we want a visualization framework that allows us to compare to data obtained from NHP PET-CT scans. We developed several updated visualizations of *HostSim* in [Figure 2](#Figure_FIGSCHEMATIC) C and Supplementary Videos 1 and 2 by obtaining a 3D triangulated mesh representing both an NHP lung surface and a NHP body from experimental data (see [Figure 2](#Figure_FIGSCHEMATIC) C). We randomly assign granulomas forming within *HostSim* locations within the volume of the right lobe of the lung. This mimics actual infection when bacteria are inhaled into the lungs, and a unique granuloma forms in response to each individual bacterial seed (24). We give locally disseminating granulomas nearby point in space and nonlocally disseminating granulomas a random point within the lung volume. As granulomas develop in *HostSim*, the evolution of their state variables and various downstream metadata are recorded, e.g., total CFU count from summing _﻿_ $B_{E}$, _﻿_$B_{I}$_﻿_﻿, and $B_{N}$_﻿_﻿. We render granulomas at each point in time along with the triangulated meshes in ParaView after conversion of the data to VTK file formats. This allows us to render the granulomas in space with variable data-defined size and color via visualization tools available in ParaView (25). The sizes of the granulomas in our visualizations are proportional to their predicted volume, as discussed in SI.

## Spatially tracking granulomas within lungs

We require *HostSim* to make predictions of the PET-CT scans of virtual hosts to compare with these data derived in NHP hosts. PET-CT scans of NHPs and humans yield “hot spots” corresponding to ^18^F-fluorodeoxyglucose (FDG) avidity that are known to be measures of metabolic activity, and one of the only measures of granuloma activity in live TB hosts (26, 27). FDG avidity data are one of the few modes of TB data that can be measured as time-series data in living TB hosts, the others being blood T-cell counts and sputum samples. The cell species whose metabolic activity primarily drives FDG avidity hot-spots are unknown, but recent unpublished data suggest that activated cells contribute (personal communication Joshua Matilla, University of Pittsburgh). If we hypothesize a set of relative cell-species contributions to FDG avidity, we can use *HostSim* to test FDG avidity predictions by calculating a weighted sum of cell counts. In granulomas, we calculate our prediction as:

$\text{Granuloma FDG avidity}=w_{1}\text{MR}+w_{2}\text{MI}+w_{3}\text{MA}+w_{4}\text{T0}+w_{5}\text{TE}+w_{6}\text{TEM}$_﻿_﻿ [S1]

where $M_{R}$﻿, $M_{I}$﻿, and $M_{A}$﻿ are resting, infected, and activated macrophages; and $T_{0}$﻿, $T_{E}$﻿, and $T_{EM}$﻿ are CD3^+^ precursor, effector, and effector memory T-cells, respectively. The weights we’ve chosen for each cell species are $w_{1} = 1$﻿ , $w_{2}=5$﻿, $w_{3}=6$﻿, $w_{4}=2$﻿, $w_{5}=4$﻿, and $w_{6}=3$, consistent with our assumption that active immune cells (e.g. $M_{A}$ and $\text{TE}$) are more metabolically active than their resting or non-differentiated counterparts. Other weights can easily be chosen to either fit data as they become available, or to test hypotheses of proposed drivers of FDG avidity. We have an analogous computation in the lymph node compartment, given by the following:

$\text{Lymph Node FDG avidity}=w_{1}\text{APC}+w_{2}\text{T0}+w_{3}\text{TE}+w_{4}\text{TEM}$﻿, [S2]

where APC is the number of antigen-presenting cells, and the notation for T-cells is the same as for granulomas. Here, we set our weights as $w_{1}=3$﻿, $w_{2}=2$﻿, $w_{3}=4$ and $w_{4}=3$﻿.

In part to make a more accurate rendering of hosts, we predict the size of granulomas within *HostSim*. Since granulomas are generally spherical in experiments (with some deviation depending on dissemination events and noise), we calculate *HostSim* geometry by assuming the granuloma is spherical, and caseum forms a smaller sphere at the center of the granuloma. Granuloma volume is computed as

$\text{Granuloma Volume}=V_{\mathrm{Mac}}\sum\left( \#M\text{Φ}+N_{f}\cdot Ca \right)+V_{\mathrm{Mtb}}\sum\#\text{CFU}+V_{\mathrm{Tcell}}\sum\#\mathrm{CD}3^{+}\text{ Cells }$_﻿_﻿.

For the first term, we assume that caseum volume comes chiefly from dead macrophages (recall that_﻿_﻿ $N_{f}$ dead macrophages make one unit of caseum in a granuloma). We use spherical volume to obtain the values $V_{\mathrm{Mac}}=4/3\pi\left( 21\mu m \right)^{3}$, $V_{\mathrm{Tcell}}=4/3\pi\left( 6\mu m \right)^{3}$; and cylindrical volume to get﻿ $V_{\mathrm{Mtb}}={\pi(0.345\mu m)}^{2}\cdot\left( 2.71\mu m \right)$.

## PRCC results of FDG avidity predictions that can be compared to other impact quantifications

The specific immunological events that promote PET-CT FDG avidity intensities *in situ* are not well-understood. With *HostSim*, we are able to investigate on a granular level the mechanistic drivers, given a proposed FDG avidity equation (see Methods). That is, given a hypothesis of the cell species that are indicated by PET-CT scans, we are able to determine what parameters (and processes) are correlated with higher FDG avidity values. We may also interrogate the biological merit of any choice of weights $w_{i}$_﻿_﻿ which prescribes the relative influence of specific cell types over FDG avidity. When applying this to our method for proposed FDG avidity expressions [1-2], we are able to isolate 14 of our 201 varied parameters that significantly impacted FDG avidity values, shown in Supplementary Table 1. Broadly speaking, we see that faster Mtb uptake into macrophages, faster macrophage recruitment, and longer-lived infected macrophages increase FDG avidity. We also see that faster differentiation of T-cells into their effector states increases FDG avidity values.

# Supplementary figures and tables


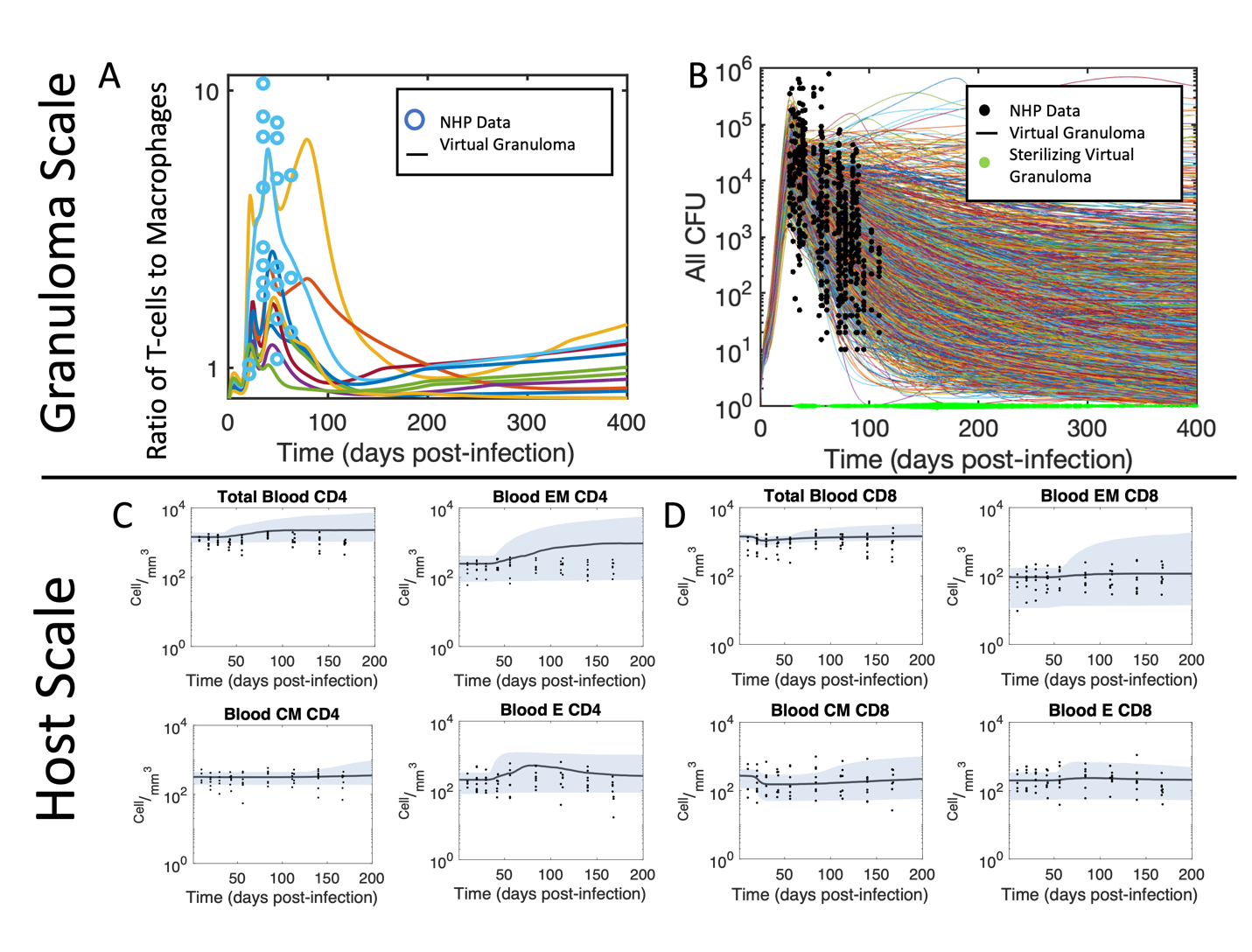


**Supplementary Figure 1**: **Host scale and Granuloma scale outputs within *HostSim***. (A) Ratio of T-cells to macrophages over time in 13 granulomas from a single virtual host are shown in curves. Circles show ratios obtained from prior work (4-7). (B) Total granuloma CFU over time are shown as curves for each primary granuloma in 500 virtual patients that did not sterilize by 400 days post-infection. NHP data are shown as black dots (4-7). Green circles along the horizontal axis represent granulomas that sterilized at that time; larger circles indicate more granulomas sterilizing on that day. (C-D) Validation: model blood concentrations of (C) CD4^+^ and (D) CD8^+^ T-cell concentrations across time, compared to NHP data from (4-7). The solid lines are the median T-cell concentrations for each cell type, and the shaded region indicates the 1st and 99th percentile for concentrations among 500 virtual hosts for each cell type.


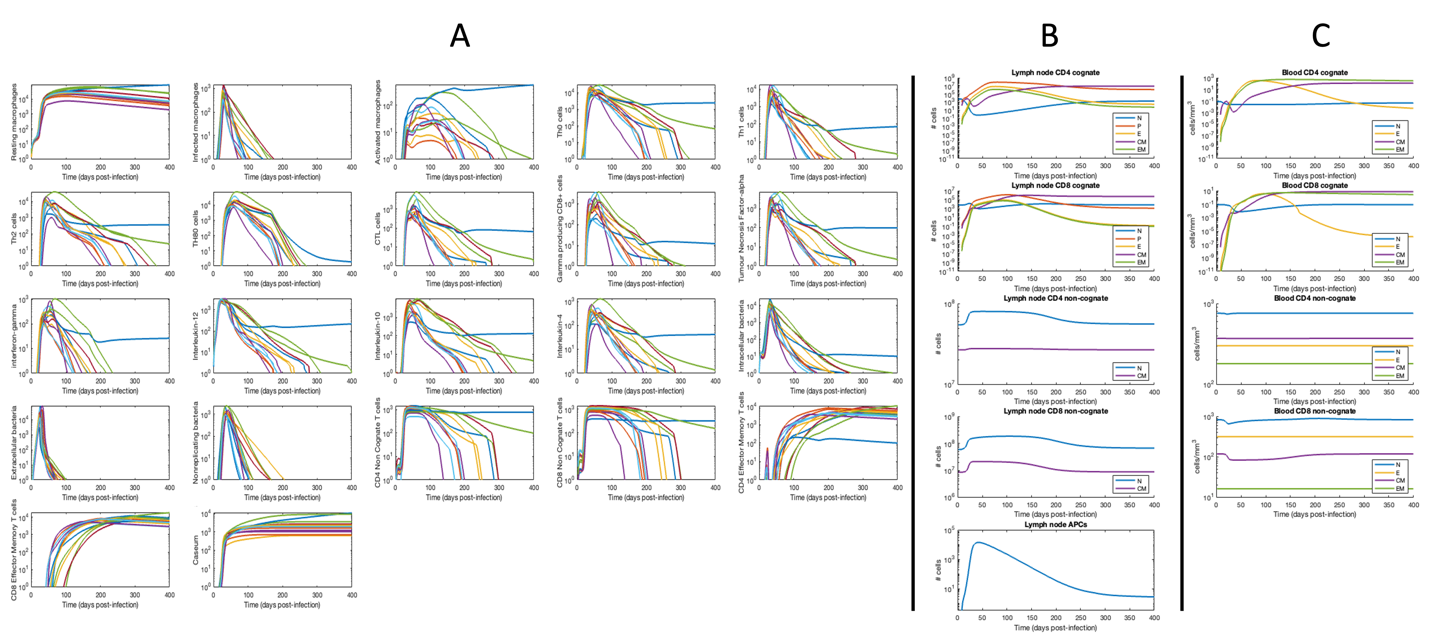


**Supplementary Figure 2: *HostSim* states for a single virtual host with clinically latent TB.** (A) Latent host granuloma-scale dynamics. Each Note that an individual granuloma has a sharp downturn in immune responses once it sterilizes, but even with one infected granuloma remaining at day 400 post-infection this virtual host remains latently infected. (B) Latent host lymph node T-cell counts. APCs are required for clonal selection to upregulate Mtb-specific CD4^+^ and CD8^+^ T-cells. (C) Latent host T-cell blood concentrations. (B-C) N indicates the naive cell type, P indicates Precursor cell type, E indicates Effector cell type, CM indicates Central Memory cell type, and EM indicates Effector Memory cell type.

| **Supplementary Table 1:** Statistically significant PRCC results for FDG avidity predictions at the granuloma and host scales over time. Indicated parameters have PRCC shown as positively or negatively correlated with FDG avidity after day 100 in with $p<0.05$_﻿_﻿. | |
| --- | --- |
| **Parameter Description** | **PRCC trend for FDG-avidity prediction** |
| **Granuloma-scale FDG avidity PRCC results** | |
| In-macrophage carrying capacity of Mtb | + |
| Resting macrophage infection rate | + |
| Precursor CD8^+^ T-cell proliferation rate in the lymph nodes | + |
| TNF-_﻿_ mediated recruitment rate of macrophages | + |
| Michaelis-Menten half-saturation of TNF-_﻿_ dependent recruitment of primed CD4^+^ T-cells | - |
| Michaelis-Menten half-saturation of Mtb uptake into macrophages | - |
| **LN-scale FDG avidity PRCC results** | |
| Precursor CD4^+^ T-cell proliferation rate in the lymph nodes | + |
| Precursor CD8^+^ T-cell proliferation rate in the lymph nodes | + |
| Michaelis-Menten half-saturation of CD4^+^ T-cell differentiation | + |
| Michaelis-Menten half-saturation of CD4^+^ T-cell proliferation | - |
| Precursor-to-effector CD4^+^ T-cell differentiation rate | - |
| Precursor-to-effector CD8^+^ T-cell differentiation rate | - |

## Supplementary References

1. Cadena AM, Fortune SM, Flynn JL. Heterogeneity in tuberculosis. Nat Rev Immunol. 2017;17(11):691-702.

2. Lin PL, Ford CB, Coleman MT, Myers AJ, Gawande R, Ioerger T, et al. Sterilization of granulomas is common in active and latent tuberculosis despite within-host variability in bacterial killing. Nature medicine. 2014;20(1):75-9.

3. Ganchua SKC, White AG, Klein EC, Flynn JL. Lymph nodes-The neglected battlefield in tuberculosis. PLoS Pathog. 2020;16(8):e1008632.

4. Gideon HP, Phuah J, Myers AJ, Bryson BD, Rodgers MA, Coleman MT, et al. Variability in tuberculosis granuloma T cell responses exists, but a balance of pro- and anti-inflammatory cytokines is associated with sterilization. PLoS pathogens. 2015;11(1):e1004603.

5. Marino S, Gideon HP, Gong C, Mankad S, McCrone JT, Lin PL, et al. Computational and Empirical Studies Predict Mycobacterium tuberculosis-Specific T Cells as a Biomarker for Infection Outcome. PLoS Comput Biol. 2016;12(4):e1004804.

6. Cadena AM, Hopkins FF, Maiello P, Carey AF, Wong EA, Martin CJ, et al. Concurrent infection with Mycobacterium tuberculosis confers robust protection against secondary infection in macaques. PLOS Pathogens. 2018;14(10):e1007305.

7. Darrah PA, DiFazio RM, Maiello P, Gideon HP, Myers AJ, Rodgers MA, et al. Boosting BCG with proteins or rAd5 does not enhance protection against tuberculosis in rhesus macaques. NPJ Vaccines. 2019;4:21.

8. Murphy BM, Singer BH, Anderson S, Kirschner D. Comparing epidemic tuberculosis in demographically distinct heterogeneous populations. Math Biosci. 2002;180:161-85.

9. Gagneux S, DeRiemer K, Van T, Kato-Maeda M, de Jong BC, Narayanan S, et al. Variable host-pathogen compatibility in Mycobacterium tuberculosis. Proc Natl Acad Sci U S A. 2006;103(8):2869-73.

10. Smith KC, Seaworth BJ. Drug-resistant tuberculosis: controversies and challenges in pediatrics. Expert Rev Anti Infect Ther. 2005;3(6):995-1010.

11. Sarathy JP, Zuccotto F, Hsinpin H, Sandberg L, Via LE, Marriner GA, et al. Prediction of Drug Penetration in Tuberculosis Lesions. ACS infectious diseases. 2016;2(8):552-63.

12. Prideaux B, Via LE, Zimmerman MD, Eum S, Sarathy J, O'Brien P, et al. The association between sterilizing activity and drug distribution into tuberculosis lesions. Nat Med. 2015;21(10):1223-7.

13. Sud D, Bigbee C, Flynn JL, Kirschner DE. Contribution of CD8+ T cells to control of Mycobacterium tuberculosis infection. J Immunol. 2006;176(7):4296-314.

14. Pienaar E, Sarathy J, Prideaux B, Dietzold J, Dartois V, Kirschner DE, Linderman JJ. Comparing efficacies of moxifloxacin, levofloxacin and gatifloxacin in tuberculosis granulomas using a multi-scale systems pharmacology approach. PLoS Comput Biol. 2017;13(8):e1005650.

15. Warsinske HC, Pienaar E, Linderman JJ, Mattila JT, Kirschner DE. Deletion of TGF-beta1 Increases Bacterial Clearance by Cytotoxic T Cells in a Tuberculosis Granuloma Model. Front Immunol. 2017;8:1843.

16. Segovia-Juarez JL, Ganguli S, Kirschner D. Identifying control mechanisms of granuloma formation during M. tuberculosis infection using an agent-based model. Journal of Theoretical Biology. 2004;231(3):357-76.

17. Budak M, Cicchese JM, Maiello P, Borish HJ, White AG, Chishti HB, et al. Optimizing tuberculosis treatment efficacy: Comparing the standard regimen with Moxifloxacin-containing regimens. PLOS Computational Biology. 2023;19(6):e1010823.

18. Mattila JT, Ojo OO, Kepka-Lenhart D, Marino S, Kim JH, Eum SY, et al. Microenvironments in tuberculous granulomas are delineated by distinct populations of macrophage subsets and expression of nitric oxide synthase and arginase isoforms. Journal of immunology. 2013;191(2):773-84.

19. Cardona PJ, Prats C. The Small Breathing Amplitude at the Upper Lobes Favors the Attraction of Polymorphonuclear Neutrophils to Mycobacterium tuberculosis Lesions and Helps to Understand the Evolution toward Active Disease in An Individual-Based Model. Frontiers in microbiology. 2016;7:354.

20. Lyadova IV. Neutrophils in Tuberculosis: Heterogeneity Shapes the Way? Mediators of inflammation. 2017;2017:8619307.

21. Hult C, Mattila JT, Gideon HP, Linderman JJ, Kirschner DE. Neutrophil Dynamics Affect Mycobacterium tuberculosis Granuloma Outcomes and Dissemination. Front Immunol. 2021;12:712457.

22. Marino S, Hult C, Wolberg P, Linderman JJ, Kirschner DE. The Role of Dimensionality in Understanding Granuloma Formation. Computation.6(4).

23. Joslyn LR, Linderman JJ, Kirschner DE. A virtual host model of Mycobacterium tuberculosis infection identifies early immune events as predictive of infection outcomes. J Theor Biol. 2022;539:111042.

24. Martin CJ, Cadena AM, Leung VW, Lin PL, Maiello P, Hicks N, et al. Digitally Barcoding Mycobacterium tuberculosis Reveals In Vivo Infection Dynamics in the Macaque Model of Tuberculosis. MBio. 2017;8(3).

25. Henderson A. The ParaView Guide: A Parallel Visualization Application: Kitware Inc.; 2007.

26. Lin PL, Coleman T, Carney JP, Lopresti BJ, Tomko J, Fillmore D, et al. Radiologic responses in cynomolgous macaques for assessing tuberculosis chemotherapy regimens. Antimicrobial agents and chemotherapy. 2013.

27. Esmail H, Lai RP, Lesosky M, Wilkinson KA, Graham CM, Coussens AK, et al. Characterization of progressive HIV-associated tuberculosis using 2-deoxy-2-[18F]fluoro-D-glucose positron emission and computed tomography. Nature Medicine. 2016;22(10):1090-3.
